# Mitochondrial glutaredoxin Grx5 functions as a central hub for cellular iron-sulfur cluster assembly

**DOI:** 10.1016/j.jbc.2025.108391

**Published:** 2025-03-10

**Authors:** Ashutosh K. Pandey, Jayashree Pain, Pratibha Singh, Andrew Dancis, Debkumar Pain

**Affiliations:** Department of Pharmacology, Physiology and Neuroscience, New Jersey Medical School, Rutgers University, Newark, New Jersey, USA

**Keywords:** cytoplasm, export, iron, iron-sulfur protein, metal cofactor, mitochondria, sulfur, tRNA thiolation, yeast

## Abstract

Iron-sulfur (Fe-S) protein biogenesis in eukaryotes is mediated by two different machineries—one in the mitochondria and another in the cytoplasm. Glutaredoxin 5 (Grx5) is a component of the mitochondrial iron-sulfur cluster machinery. Here, we define the roles of Grx5 in maintaining overall mitochondrial/cellular Fe-S protein biogenesis, utilizing mitochondria and cytoplasm isolated from *Saccharomyces cerevisiae* cells. We previously demonstrated that isolated wild-type (WT) mitochondria themselves can synthesize new Fe-S clusters, but isolated WT cytoplasm alone cannot do so unless it is mixed with WT mitochondria. WT mitochondria generate an intermediate, called (Fe-S)_int_, that is exported to the cytoplasm and utilized for cytoplasmic Fe-S cluster assembly. We here show that mitochondria lacking endogenous Grx5 (Grx5↓) failed to synthesize Fe-S clusters for proteins within the organelle. Similarly, Grx5↓ mitochondria were unable to synthesize (Fe-S)_int_, as judged by their inability to promote Fe-S cluster biosynthesis in WT cytoplasm. Most importantly, purified Grx5 precursor protein, imported into isolated Grx5↓ mitochondria, rescued these Fe-S cluster synthesis/trafficking defects. Notably, mitochondria lacking immediate downstream components of the mitochondrial iron-sulfur cluster machinery (Isa1 or Isa2) could synthesize [2Fe-2S] but not [4Fe-4S] clusters within the organelle. Isa1↓ (or Isa2↓) mitochondria could still support Fe-S cluster biosynthesis in WT cytoplasm. These results provide evidence for Grx5 serving as a central hub for Fe-S cluster intermediate trafficking within mitochondria and export to the cytoplasm. Grx5 is conserved from yeast to humans, and deficiency or mutation causes fatal human diseases. Data as presented here will be informative for human physiology.

Glutaredoxins (Grxs) belong to the thioredoxin superfamily, and they can be classified primarily into two groups (1, 2, 3, 4). Dithiol Grxs (class I) with a Cys-Pro-[Tyr/Phe]-Cys active site motif are oxidoreductases; they catalyze oxidation and reduction of protein disulfides and also mixed disulfides between protein thiols and GSH. The monothiol Grxs (class II) share a consensus Cys-Gly-Phe-Ser (CGFS) active site motif that contains a thiol at its N terminus but lacks another thiol at its C terminus. Unlike class I Grxs, class II Grxs do not usually exhibit oxidoreductase activity. Instead, class II Grxs have been implicated in iron metabolism and iron-sulfur (Fe-S) cluster assembly (1, 2, 3, 4).

In the yeast *Saccharomyces cerevisiae*, Grx5 is a single domain Cys-Gly-Phe-Ser Grx that is localized in mitochondria (5). Two Grx5 molecules are bridged by a [2Fe-2S]^2+^ cluster that is coordinated symmetrically by four Cys ligands: two active-site cysteine residues and two GSH molecules that are noncovalently attached to the protein in a specific pocket (4, 6, 7, 8, 9). The protein is conserved from yeast to zebrafish to humans, and its deficiency is associated with dysregulated cellular iron homeostasis such as diminished mitochondrial and/or cytoplasmic Fe-S enzyme activities, impaired heme synthesis, mitochondrial iron overload, oxidative damage, and relative cytoplasmic iron depletion (1, 5, 10, 11, 12, 13, 14). These features are often considered signature phenotypes associated with defects in Fe-S cluster assembly in mitochondria and/or cytoplasm (15, 16). However, the Grx5 functions in these compartmentalized processes are not fully understood.

Fe-S clusters are inorganic protein cofactors, with the most prominent examples being [2Fe-2S] and [4Fe-4S] types. Fe-S proteins play vital roles in fundamental cellular processes, including respiration, enzyme catalysis, DNA synthesis and repair, tRNA modifications, protein translation, and sensing of intracellular oxygen or iron levels (17, 18, 19). In eukaryotes, Fe-S proteins are found in mitochondria as well as outside of mitochondria, mostly in cytoplasm and nucleus. The biogenesis of mitochondrial Fe-S proteins in yeast can be broadly divided into three main steps (16, 20, 21). The first step involves *de novo* synthesis of a [2Fe-2S] cluster on a scaffold protein Isu1 (or the Isu2 isoform). The Nfs1 cysteine desulfurase forms an active enzyme complex with its regulatory partners (Isd11 and Acp1) and provides sulfur from the amino acid cysteine. Frataxin might be involved in promoting persulfide sulfur formation on Nfs1 and/or its transfer to Isu1 (22, 23, 24, 25, 26). The Fe-S cluster synthesis on Isu1 also requires the redox couple ferredoxin/ferredoxin reductase (Yah1/Arh1) that likely reduces sulfane sulfur to sulfide (27, 28, 29, 30). The precise source and/or form of iron in the mitochondrial matrix for Fe-S cluster synthesis has not yet been determined (31). In the second step, the newly assembled [2Fe-2S] cluster intermediate on Isu1 is transferred to Grx5 in a process requiring the Hsp70 chaperone Ssq1, cochaperone Jac1, and the ADP-ATP exchange factor Mge1. In the third step, Grx5 is thought to deliver its [2Fe-2S] cluster to the ISA complex (Isa1/Isa2/Iba57) for [4Fe-4S] cluster synthesis (32, 33, 34). The newly formed [4Fe-4S] cluster is then transferred to apoproteins (*e.g.*, aconitase), and this transfer process may or may not require additional proteins such as Nfu1, Bol1, and Bol3 depending on target proteins (16, 20, 35).

More in depth study of the Fe-S cluster donation role of Grx5 was accomplished by Lill and co-workers in biochemical reconstitution assays (35). They were able to reconstitute [4Fe-4S] cluster loading of human aconitase (ACO2) using the [2Fe-2S]-containing GLRX5 (the human homolog of yeast Grx5) as the cluster donor. The process required ISA1, ISA2, and IBA57 proteins. Additionally, mitochondrial ferredoxin FDX2 and its reductase FDXR were also needed for reductive fusion of [2Fe-2S] into [4Fe-4S] clusters. The transfer of [2Fe-2S] cluster from GLRX5 to other [2Fe-2S] recipient proteins, however, occurred spontaneously without requiring any mitochondrial iron-sulfur cluster (ISC) machinery components (35).

The biogenesis of Fe-S proteins residing in the cytoplasm and nucleus requires additional steps. The ISC machinery, including the Nfs1 cysteine desulfurase complex, Isu1/2 scaffold, chaperones, and up to Grx5 (see Fig. 8*B*), mediates synthesis of an intermediate called X-S or (Fe-S) intermediate (Fe-S)_int_. The intermediate is then exported from the mitochondrial matrix to the cytoplasm *via* Atm1, an ABC transporter in the mitochondrial inner membrane (16, 20). Once outside the mitochondria, the exported intermediate is utilized by a separate but parallel machinery, called cytoplasmic iron-sulfur protein assembly (CIA), undergoing another round of scaffold binding, reductase activity and assembly in the cytoplasm, generating active Fe-S proteins (16, 17). Of note, the (Fe-S)_int_ donated (directly or indirectly) by Grx5 to Atm1 still needs purification, identification, chemical characterization, and only then this mitochondria–cytoplasm interaction/communication in Fe-S cluster assembly can perhaps be fully reconstituted. At this stage, the intermediate appears to contain iron, sulfur and GSH, perhaps as a [2Fe-2S](GS)_4_ compound (36).

Lill and co-workers developed an important assay to study Fe-S protein biogenesis in yeast cells. The assay involves *in vivo* ^55^Fe labeling of whole cells, recovery of the target Fe-S proteins by immunoprecipitation or affinity pull-down, and measurement of radioactivity (37). This approach often requires overproduction of target Fe-S proteins to enhance the ^55^Fe detection limit. The method has been used to investigate the roles of various ISC (and CIA) components. For example, when Grx5-depleted cells were labeled with ^55^Fe nuclide, the radiolabel associated with marker Fe-S proteins in mitochondria and cytoplasm was found to be decreased (38). On the other hand, ^55^Fe associated with Isu1, possibly as ^55^Fe-S cluster, was markedly increased. Likewise, for ssq1 and jac1 mutants, subjected to similar ^55^Fe labeling, the recovered radioactivity was decreased for target Fe-S proteins and increased for the Isu1/2 intermediates. These results led to the suggestion that Grx5, like Ssq1 and Jac1, is required for a transfer step following Fe-S cluster synthesis on the Isu1 scaffold (39), and thus in ssq1, jac1 or grx5 mutants, the ^55^Fe “backs up” on the Isu1/2 precursor intermediate prior to the transfer/utilization step (see Fig. 8*B*).

We found that mitochondria, as isolated from WT cells, contain a complete ISC machinery and can form new Fe-S clusters when supplemented with cysteine, iron, and nucleotides. The newly formed [2Fe-2S] and [4Fe-4S] clusters are efficiently inserted into appropriate apoproteins in the mitochondrial matrix (40, 41). Isolated WT cytoplasm by itself, however, cannot synthesize Fe-S clusters. Notably, the biosynthetic process for formation of new cytoplasmic Fe-S clusters proceeds when mitochondria are added back to the cytoplasm (42, 43, 44). Here, we used an experimental setup involving mitochondria alone, cytoplasm alone, or mitochondria-cytoplasm mixing to define the role of Grx5 in Fe-S cluster formation/trafficking. An exciting finding is that newly imported Grx5 protein was able to reestablish Fe-S cluster assembly in mitochondria lacking endogenous Grx5, with concomitant restoration of the mitochondrial ability to promote cytoplasmic Fe-S cluster biogenesis. We presume that the restored mitochondria synthesize the (Fe-S)_int_, which must be exported from mitochondria to cytoplasm for cytoplasmic Fe-S cluster assembly to occur. These results ascertain a decisive role of Grx5 in Fe-S cluster trafficking in mitochondria that is needed for overall cellular Fe-S protein homeostasis.

## Results

### Evaluating Grx5↓ and other downstream mutants of the ISC machinery for effects on [4Fe-4S] cluster biogenesis of aconitase

To define the role of Grx5 in mitochondria/cellular Fe-S cluster biogenesis, we began with analyzing the phenotypes associated with cellular depletion of Grx5. We generated a yeast strain in which Grx5 expression can be turned on or off by changing medium composition. Briefly, the native promoter for Grx5 was replaced with the *GAL1* promoter in the genome (Gal-Grx5) using the Longtine method (45). The parent BY4741 served as congenic WT strain. Similarly, other yeast strains were generated with regulated expression of the downstream ISA components Isa1, Isa2, or Iba57. Deletion strains of further downstream ISC components, such as Δnfu1 or Δbol3, were used as specificity controls. All cells were grown in raffinose plus dextrose medium. This is a repressing condition for the swapped *GAL1* promoter in the Gal strains. Mitochondria were isolated from these cells in an enriched preparation (Fig. S1) and evaluated for Fe-S cluster biogenesis of aconitase as described below. We previously did similar studies with Grx5 upstream components of the ISC machinery (42, 43, 44), but here the focus is on Grx5 (and some downstream components).

**Figure 1:**
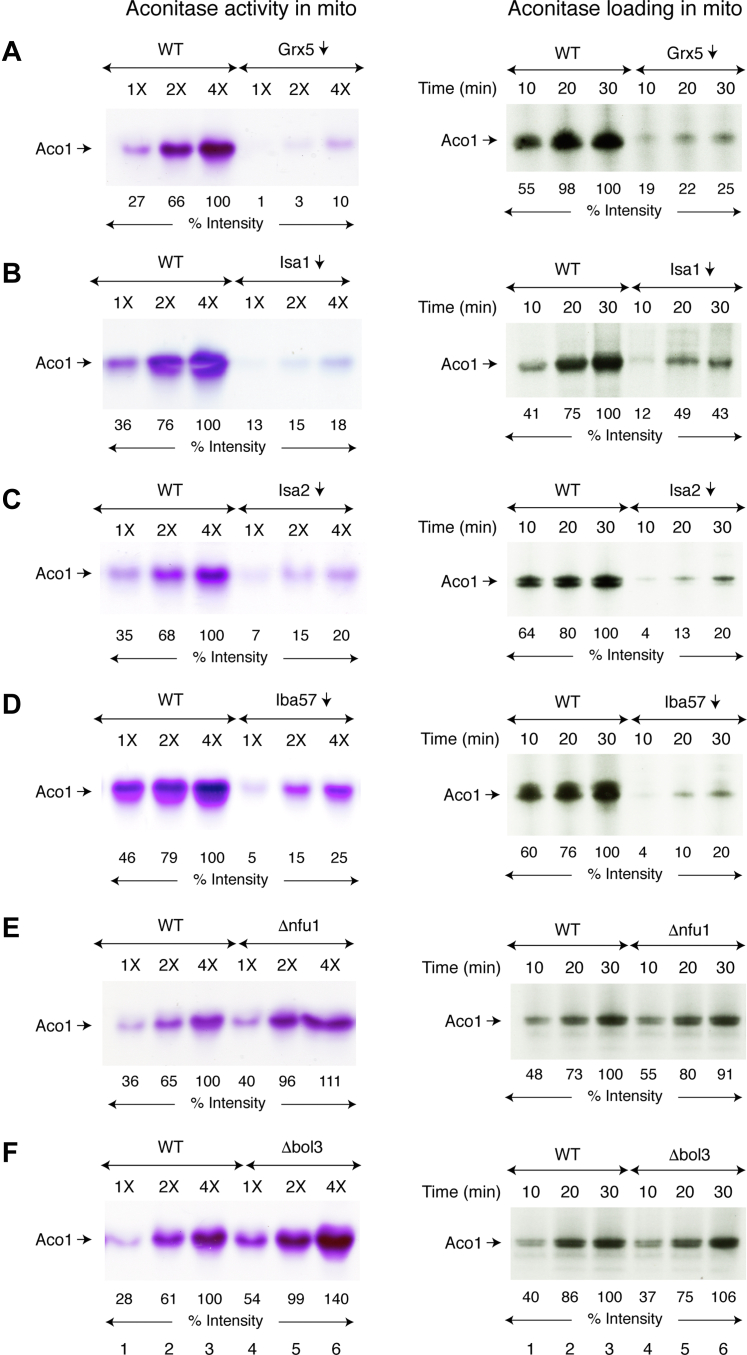
**Correlating enzyme activity and [4Fe-4S] cluster loading of endogenous aconitase in isolated mitochondria.** The native promoter of several genes (*GRX5*, *ISA1*, *ISA2*, and *IBA57*) was individually replaced with the *GAL1* promoter in the genome (45), generating corresponding Gal strains (Table S1). The WT BY4741, Gal strains, and two deletion strains (Δnfu1 and Δbol3) were grown in raffinose plus dextrose medium (no galactose), and mitochondria were isolated. Under these conditions, the *GAL1* promoter in Gal strains is turned off and expression of the corresponding protein is repressed (↓). *A*–*F*, *left panels*: isolated mitochondria (“mito”) were lysed, subjected to native PAGE, and analyzed for aconitase activity by an in-gel assay (44, 54). 1X = 50 μg of proteins. *A*–*F*, *right panels*: WT or mutant mitochondria (200 μg of proteins) were incubated with [^35^S]cysteine (10 μCi), nucleotides (4 mM ATP, 1 mM GTP, 2 mM NADH), and ferrous ascorbate (10 μM) at 30 °C for 10 to 30 min. Reaction mixtures were diluted with isotonic buffer and centrifuged. The mitochondrial pellets thus obtained were analyzed by native PAGE, followed by autoradiography (40, 42). Grx5, glutaredoxin 5.

Aconitase [4Fe-4S] is an important enzyme involved in the tricarboxylic acid cycle in mitochondria. The enzyme reversibly catalyzes the isomerization of citrate to isocitrate, and this activity is strictly dependent on its Fe-S cluster. Aconitase (Aco1) activity in mitochondria isolated from various strains was measured by a native in-gel assay (Fig. 1, *A*–*F*, left panels, “aconitase activity in mitochondria”). Practically no aconitase activity was detected in mitochondria isolated from Gal-Grx5 repressed (*i.e.*, Grx5-depleted; Grx5↓) cells (Fig. 1*A*, left panel, lanes 4–6). Aco1 activity was also virtually undetectable in Isa1↓ mitochondria (Fig. 1*B*, left panel). Similarly, depletion of other components of the ISA complex (Isa2↓ or Iba57↓) was associated with significantly reduced Aco1 activity (Fig. 1, *C* and *D* left panels, respectively). In contrast, mitochondria lacking Nfu1 (Fig. 1*E*, left panel) or Bol3 (Fig. 1*F*, left panel) exhibited WT levels of Aco1 activity. Similar data from a different set of experiments are presented in Fig. S2, *A* and *B*, left panels), showing reproducibility. These results confirmed expected Aco1 activity phenotypes (5, 32, 33, 34, 35), prompting us to evaluate if these isolated mitochondria can synthesize/assemble [4Fe-4S] clusters for Aco1 as follows.

In our assays with isolated mitochondria, [^35^S]cysteine was used as the source of radiolabeled sulfur, and insertion of newly formed Fe-^35^S clusters into apoproteins was monitored. Briefly, mitochondria were supplemented with [^35^S]cysteine, nucleotides (ATP, GTP, and NADH), and ferrous ascorbate as iron supplier. After incubation at 30 °C for 10 to 30 min, mitochondria were reisolated by centrifugation and analyzed by native PAGE, followed by autoradiography to directly visualize radiolabeled proteins (Fig. 1, *A*–*F*, right panels, “aconitase loading in mitochondria”). The Fe-^35^S clusters are destroyed by denaturants such as SDS, and thus, radiolabeled Fe-S proteins can only be analyzed by native gels.

In WT mitochondria, endogenous Aco1 was found to be strongly radiolabeled, most likely due to insertion of newly made [4Fe-4^35^S] clusters (Fig. 1*A*, right panel, lanes 1–3). In contrast, Grx5-depleted (Grx5↓) mitochondria exhibited very little radiolabeling of aconitase, implying deficient [4Fe-4^35^S] cluster biogenesis (Fig. 1*A*, right panel, lanes 4–6; and Fig. S2*A*, right panel). Similarly, mitochondria lacking any of the components of the ISA complex (Isa1, Isa2, or Iba57) produced greatly reduced levels of radiolabeled Aco1 (Fig. 1, *B*–*D*, right panels). The Δnfu1 and Δbol3 mitochondria, however, behaved just like WT mitochondria in terms of Aco1 radiolabeling (Fig. 1, *E* and *F*, right panels; and Fig. S2*B*, right panel). These results show that Grx5 and ISA components indeed play critical roles for new synthesis and/or insertion of [4Fe-4S] clusters into Aco1 in our assays with isolated and intact mitochondria. Aconitase and the Nfs1 cysteine desulfurase protein levels in various mutant mitochondria were comparable to WT levels (Fig. S3). Thus, poor radiolabeling of Aco1 in Grx5↓ mitochondria or ISA mutant mitochondria likely reflects deficient [4Fe-4S] cluster synthesis/assembly rather than lack of Aco1 protein. This issue is further addressed below.

### Restoring aconitase loading in Grx5↓ or Isa1↓ mitochondria by importing corresponding proteins

To determine the specific roles of Grx5 and Isa1 in mitochondrial Fe-S cluster biogenesis, we tested if Grx5 or Isa1 imported into mitochondria isolated from Grx5-depleted or Isa1-depleted cells can restore [4Fe-4S] cluster loading of aconitase. Briefly, the precursor forms of Grx5 and Isa1 proteins with a C-terminal His_6_ tag were individually expressed in bacteria. In both cases, the proteins were found to be mostly sequestered in inclusion bodies. The proteins were solubilized with urea and centrifuged. The supernatant fractions contained mostly (∼80–90%) homogenous precursor proteins (Fig. S4). These preparations were used for mitochondrial protein import and simultaneous Fe-S cluster loading of endogenous Aco1 as described below. Note that import of urea-denatured precursor proteins does not require addition of cytoplasmic chaperones. The urea-denatured precursor protein circumvents a rate-limiting step such as unfolding of the native precursor protein prior to import into isolated mitochondria (46, 47). Consequently, import usually occurs faster and with high efficiency. Further, protein import and Fe-S cluster loading conditions are very similar, both requiring nucleotides (42, 48).

**Figure 2:**
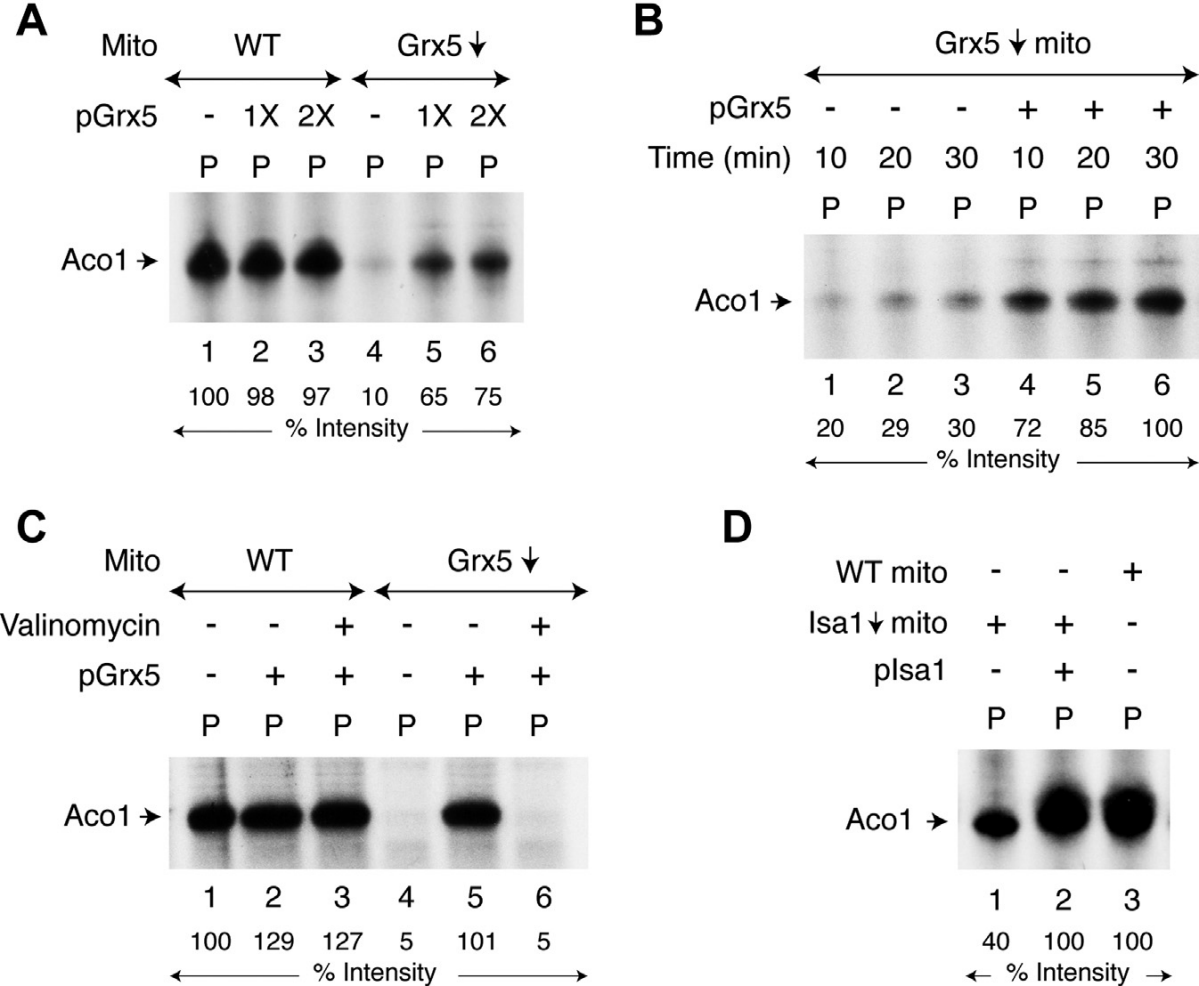
**Effects of newly imported Grx5 or Isa1 into isolated mitochondria on endogenous aconitase [4Fe-4S] cluster loading.***A*, WT or Grx5-depleted (Grx5↓) mitochondria (“Mito”; 200 μg of proteins) were supplemented with [^35^S]cysteine, nucleotides, iron, and as indicated, purified Grx5 precursor protein (pGrx5; 1X = 0.5 μg). Samples were incubated at 30 °C for 30 min, centrifuged, and mitochondrial pellets (“P”) were analyzed by native PAGE, followed by autoradiography. *B*, Grx5↓ mitochondria (200 μg of proteins) were supplemented with [^35^S]cysteine, nucleotides, and iron, and then incubated at 30 °C for 10 to 30 min, with or without added pGrx5 protein (1 μg) as indicated. Samples were analyzed as in (*A*) above. *C*, mitochondria (WT or Grx5↓; 200 μg of proteins) were incubated on ice for 2 min, with or without valinomycin (5 μM). Assay mixtures were then supplemented with [^35^S]cysteine, nucleotides, iron, and as indicated, pGrx5 protein (1 μg). After incubation at 30 °C for 30 min, samples were analyzed as in (*A*) above. *D*, WT or Isa1-depleted (Isa1↓) mitochondria (200 μg of proteins) were supplemented with [^35^S]cysteine, nucleotides, iron and as indicated, purified Isa1 precursor protein (pIsa1; 1 μg). Samples were incubated at 30 °C for 30 min and subsequently analyzed as in (*A*) above. Grx5, glutaredoxin 5; pGrx5, Grx5 precursor protein.

Briefly, isolated mitochondria (WT or Grx5↓) were supplemented with [^35^S]cysteine, nucleotides (ATP, GTP, and NADH), and ferrous ascorbate. Samples were incubated with or without added Grx5 precursor protein (pGrx5). Reaction mixtures were diluted with isotonic buffer, centrifuged, and the resulting mitochondrial pellets (“P”) were analyzed by native PAGE, followed by autoradiography. As expected, Aco1 was found to be radiolabeled with a newly made Fe-^35^S cluster in WT mitochondria, regardless of added pGrx5 (Fig. 2*A*, lanes 1–3). Practically no such radiolabeling was observed with Grx5↓ mitochondria alone (Fig. 2*A*, lane 4). Notably, however, addition of pGrx5 to Grx5↓ mitochondria led to significant radiolabeling of Aco1 (Fig. 2*A*, lanes 5 and 6) and the signal increased with incubation time (Fig. 2*B*). These results suggest that pGrx5 was imported into Grx5↓ mitochondria and that the imported molecules subsequently corrected the defects in these mutant mitochondria, thereby restoring Fe-S cluster biogenesis of Aco1. To further substantiate this notion, similar assays were performed with valinomycin-treated mitochondria. No radiolabeling of Aco1 was observed when pGrx5 was added to Grx5↓ mitochondria that had been pretreated with valinomycin (Fig. 2*C*, compare lanes 5 and 6). Valinomycin likely dissipated mitochondrial membrane potential (Δψ), blocked pGrx5 import into the matrix, and consequently, the Fe-S cluster loading defects in Grx5↓ mitochondria persisted. In contrast, Fe-S cluster assembly in WT mitochondria (endogenous Grx5 present) occurred with or without added/imported pGrx5 and was not affected by valinomycin treatment (Fig. 2*C*, lanes 1–3). The ability of isolated mitochondria to import pGrx5 was confirmed using [^35^S]methionine-labeled precursor protein and subsequent analysis of the samples by SDS-PAGE and autoradiography. Upon import, the mitochondrial targeting signal of pGrx5 was removed by matrix processing peptidases, generating the corresponding mature protein (mGrx5; Fig. S5) (5). Similarly, the Isa1 precursor protein (pIsa1) imported into isolated Isa1↓ mitochondria greatly stimulated Fe-^35^S labeling of Aco1 (Fig. 2*D*, compare lanes 1 and 2). In summary, the Aco1 [4Fe-4S] cluster assembly defects in Grx5↓ or Isa1↓ mitochondria were markedly corrected by importing Grx5 or Isa1, respectively. Thus, the observed Fe-S cluster assembly defects in our assays with isolated Grx5↓ or Isa1↓ mitochondria are most likely due to lack of Grx5 or Isa1, and not due to secondary effects that might have occurred during *in vivo* depletion of these proteins. Note that the null strains Δgrx5 and Δisa1 (or Δisa2, Δiba57) often exhibit pleiotropic secondary phenotypes including mitochondrial iron accumulation, oxidative damage to proteins, and loss of mitochondrial DNA (5, 49). These defects cannot be reversed by newly imported proteins in isolated mitochondria.

### Grx5, but not Isa1, required for new [2Fe-2S] cluster assembly in mitochondria

A prerequisite for [4Fe-4S] cluster biogenesis is the synthesis/assembly of [2Fe-2S] clusters, which are reductively coupled to generate [4Fe-4S] clusters (16, 20). Both Grx5 and Isa1 being specifically needed for [4Fe-4S] cluster assembly of Aco1 (Fig. 2), we sought to determine if they are also needed for prior [2Fe-2S] cluster assembly in our assays with isolated mitochondria. Ferredoxin (Yah1) contains a [2Fe-2S] cluster and like Aco1, the Yah1 protein is also present in the mitochondrial matrix. Unlike Aco1, however, Yah1 is much less abundant and Fe-^35^S labeling of endogenous Yah1 was not observed. This limitation could be overcome by importing Yah1 into isolated mitochondria (40, 50). Upon import into mitochondria, the targeting signal of the Yah1 precursor protein (pYah1) was cleaved by matrix processing peptidases. The mature form of Yah1 thus generated could act as a substrate for [2Fe-2S] cluster loading in the matrix. The pYah1 protein was expressed in bacteria and the inclusion bodies containing the protein were solubilized with urea to obtain apo-pYah1 for import and subsequent Fe-S cluster loading as follows.

**Figure 3:**
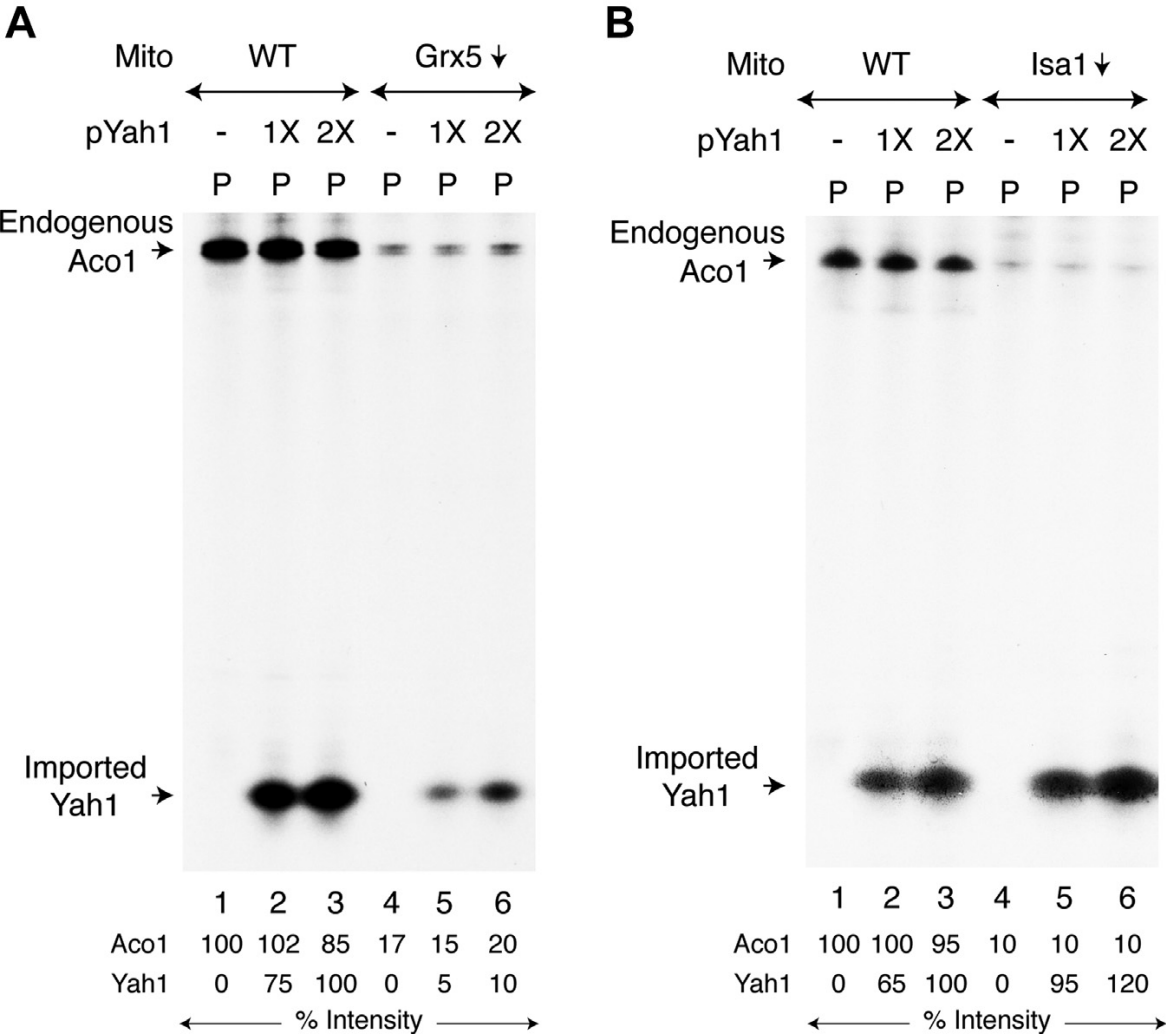
**Effects of Grx5 depletion or Isa1 depletion on [2Fe-2S] cluster loading of newly imported ferredoxin (Yah1) in isolated mitochondria.***A*, WT or Grx5↓ mitochondria (200 μg of proteins) were supplemented with [^35^S]cysteine, nucleotides, iron and as indicated, ferredoxin precursor protein (pYah1; 1X = 0.5 μg) (40). Samples were incubated at 30 °C for 30 min, centrifuged, and mitochondrial pellets (“P”) were analyzed by native PAGE, followed by autoradiography. *B*, assays were performed with WT and Isa1↓ mitochondria under identical conditions as in (*A*) above. Grx5, glutaredoxin 5; pYah1, Yah1 precursor protein.

Isolated mitochondria (WT or Grx5↓) were supplemented with [^35^S]cysteine, nucleotides, and iron. Samples were incubated with or without apo-pYah1 protein, centrifuged, and the mitochondrial pellets (“P”) were analyzed by native PAGE, followed by autoradiography. In WT mitochondria with no pYah1 added, only endogenous Aco1 was found to be radiolabeled with Fe-^35^S clusters (Fig. 3*A*, lane 1). When pYah1 was included in the assay, a faster migrating and strongly radiolabeled band (in addition to radiolabeled Aco1) appeared in WT mitochondria (Fig. 3*A*, lanes 2 and 3). This additional band corresponds to the mature form of imported Yah1 that became radiolabeled with newly formed and inserted [2Fe-2^35^S] clusters. No such Yah1 radiolabeling was observed when WT mitochondria were pretreated with carbonyl cyanide m-chlorophenyl hydrazone or valinomycin to dissipate the mitochondrial membrane potential, thereby blocking pYah1 import. Under these conditions, however, Fe-^35^S clusters continued to be made and inserted into endogenous Aco1 (Fig. S6).

Similar experiments were then performed with Grx5↓ (Fig. 3*A*) or Isa1↓ (Fig. 3*B*) mitochondria. In both cases, endogenous Aco1 radiolabeling was practically undetectable. Compared to WT mitochondria, radiolabeling of imported Yah1 occurred very poorly in Grx5↓ mitochondria (Fig. 3*A*, compare lanes 2 and 3 with lanes 5 and 6, respectively). Strikingly, however, the [2Fe-2^35^S] signal for imported Yah1 in Isa1↓ mitochondria appeared as strong as in WT mitochondria (Fig. 3*B*, compare lanes 2 and 3 with lanes 5 and 6, respectively). Thus, Grx5 and Isa1 appear to be involved in distinct stages of Fe-S cluster assembly in our assays with isolated mitochondria. Whereas Grx5 and Isa1 both are required for overall Aco1 [4Fe-4S] cluster biogenesis, Grx5 but not Isa1 is necessary for earlier stages involving [2Fe-2S] cluster synthesis/loading of Yah1. Grx5 likely plays a critical role in trafficking [2Fe-2S] clusters/intermediates to various recipients such as Yah1, Isa1, and perhaps others within mitochondria.

### Grx5, but not ISA components, required for new Fe-S cluster assembly in cytoplasm

We have recently developed mitochondria-cytoplasm mixing assays to define the role of mitochondrial ISC in cytoplasmic biosynthetic processes. We found that isolated cytoplasm by itself cannot synthesize Fe-S clusters. Addition of mitochondria to the cytoplasm, however, allows this process to proceed (42, 43, 44). Similarly, thiolation of tRNAs is critical for accurate protein synthesis (51, 52, 53), and isolated cytoplasm alone cannot thiolate tRNAs but will do so upon addition of mitochondria (42, 54). A typical experimental set up is as follows (Fig. S7). When WT mitochondria were incubated with [^35^S]cysteine, nucleotides, and iron, Fe-^35^S clusters were made and inserted into endogenous aconitase (Aco1) (Fig. S7*A*, lane 1). When WT cytoplasm was likewise incubated, there was no signal at all (Fig. S7*A*, lane 2). WT mitochondria were then added to WT cytoplasm and incubated with [^35^S]cysteine. A radiolabeled band was now detected in the cytoplasm, and this was due to ^35^S-thiolation of endogenous tRNAs (Fig. S7*A*, lane 4) as it was sensitive to RNase digestion (54). However, no radiolabeling of an endogenous cytosolic protein (analogous to endogenous Aco1 in mitochondria) was detected that might represent an Fe-S protein in the cytoplasm.

We therefore used an N-terminal truncated form of apoferredoxin (apo-ΔNYah1) as an indicator substrate for cytoplasmic Fe-S cluster assembly. The protein with a C-terminal His_6_ tag was expressed in bacteria in soluble form, purified, and converted to the apo-form by acid treatment (42). The apoprotein was incubated with cytoplasm alone in the presence of [^35^S]cysteine, nucleotides, and iron, but there was no signal (Fig. S7*B*, lane 4). Only when mitochondria were added to cytoplasm, ΔNYah1 became radiolabeled, similar to the thiolated tRNAs (Fig. S7*B*, lane 2). The radiolabeling of ΔNYah1 was most likely due to insertion of a newly formed cytoplasmic [2Fe-2^35^S] cluster into the protein. Note that the ΔNYah1 protein lacks the N-terminal mitochondrial targeting signal and therefore, it cannot enter mitochondria and remains in the cytoplasm. These results suggest that mitochondria generate and export critical sulfur-containing intermediates that are used by the cytoplasm for tRNA thiolation and Fe-S cluster assembly.

In subsequent studies (42), we found that the ISC machinery in mitochondria generates two distinct intermediates, S_int_ and (Fe-S)_int_. These intermediates are exported to the cytoplasm by the Atm1 transporter in the mitochondrial inner membrane. Once exported, S_int_ is utilized for tRNA thiolation by a sulfur relay in the cytoplasm and (Fe-S)_int_ is utilized by the CIA machinery for cytoplasmic Fe-S cluster assembly. Notably, the S_int_ synthesis requires “early” ISC components such as the Nfs1 cysteine desulfurase, its partner proteins (Isd11 and Acp1), and Isu1/2 scaffold but not a “later” component such as the Ssq1 chaperone. In contrast, (Fe-S)_int_ synthesis requires all of these proteins (42, 43, 44). Isu1/2 scaffold may constitute a branch point with one mutant allele allowing formation of S_int_ but not (Fe-S)_int_ (42). Ssq1 downstream ISC components (*e.g.*, Grx5, ISA complex, and others) might provide another branch point, with mutants allowing (Fe-S)_int_ formation but not [4Fe-4S] cluster assembly of mitochondrial aconitase. We tested this hypothesis directly as follows.

**Figure 4:**
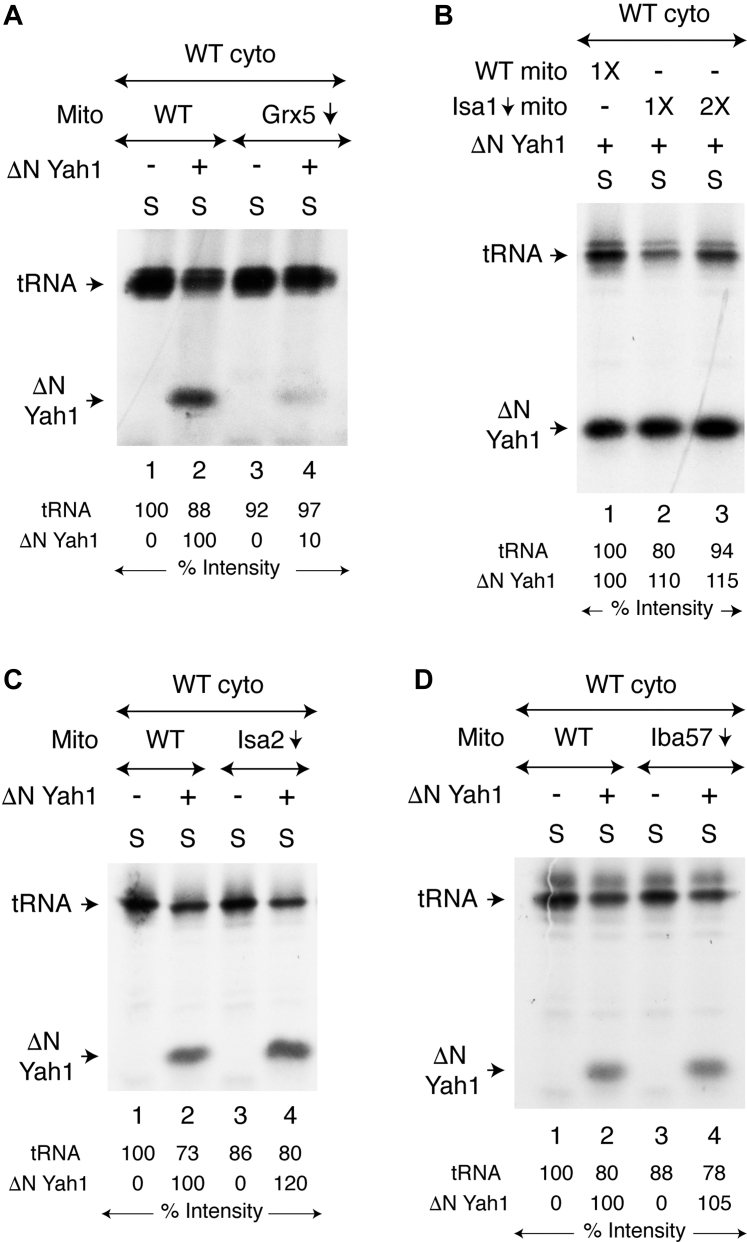
**Presence of Grx5, but not ISA components, in mitochondria needed for promoting [2Fe-2S] cluster assembly in isolated WT cytoplasm.***A*, mitochondria (WT or Grx5↓; 200 μg of proteins) were mixed with isolated WT cytoplasm (200 μg of proteins), and reaction mixtures were supplemented with [^35^S]cysteine (10 μCi), nucleotides (4 mM ATP, 1 mM GTP, 2 mM NADH), iron (10 μM) and as indicated, apo-ΔNYah1 protein (1 μg) (42). Samples were incubated at 30 °C for 30 min and centrifuged. The cytoplasm/supernatant (“S”) fractions thus obtained were analyzed by native PAGE, followed by autoradiography. *B*, mitochondria (WT or Isa1↓; 1X = 100 μg of proteins) were mixed with WT cytoplasm (200 μg of proteins), and reaction mixtures were supplemented with [^35^S]cysteine, nucleotides, iron, and apo-ΔNYah1 protein. Assays were performed as in (*A*) above. *C*, mitochondria (WT or Isa2↓; 200 μg of proteins) were mixed with WT cytoplasm (200 μg of proteins), and reaction mixtures were supplemented with [^35^S]cysteine, nucleotides, iron and as indicated, apo-ΔNYah1 protein. Assays were performed as in (*A*) above. *D*, mitochondria (WT or Iba57↓; 200 μg of proteins) were mixed with WT cytoplasm (200 μg of proteins). Samples were supplemented with [^35^S]cysteine, nucleotides, iron and as indicated, apo-ΔNYah1 protein. Assays were performed as in (*A*) above. ΔNYah1, N-terminal 60 amino acids including the mitochondrial targeting signal removed from the Yah1 precursor protein (pYah1); Grx5, glutaredoxin 5.

Isolated mitochondria (WT or Grx5↓) were mixed with isolated WT cytoplasm. Samples were incubated with [^35^S]cysteine, nucleotides, and iron, in the absence or presence of added ΔNYah1 protein. Mitochondria were then removed by centrifugation, and the resulting cytoplasm/supernatant (“S”) fractions were analyzed by native PAGE, followed by autoradiography. Grx5↓ mitochondria generated strong cytoplasmic ^35^S-labeled tRNA signals as efficiently as observed with WT mitochondria, regardless of added ΔNYah1 (Fig. 4*A*). In contrast, a strong Fe-^35^S–labeled ΔNYah1 signal was observed only with WT mitochondria and not with Grx5↓ mitochondria (Fig. 4*A*, compare lanes 2 and 4). Similar experiments were then performed with mitochondria lacking components of the ISA complex, that is, Isa1↓, Isa2↓, or Iba57↓ mitochondria (Fig. 4, *B*–*D*, respectively). Strikingly, they all were able to generate *both* radiolabeled tRNA and radiolabeled ΔNYah1 efficiently, and the corresponding signals were comparable to those observed with WT mitochondria. Whereas Grx5 in mitochondria is not needed for S_int_ production for cytoplasmic tRNA thiolation, it appears to play a critical role for (Fe-S)_int_ production for cytoplasmic [2Fe-2S] cluster assembly. The Grx5 downstream components of the ISC machinery (*i.e.*, the ISA complex) are not required for the production of S_int_ or (Fe-S)_int_ within mitochondria.

**Figure 5:**
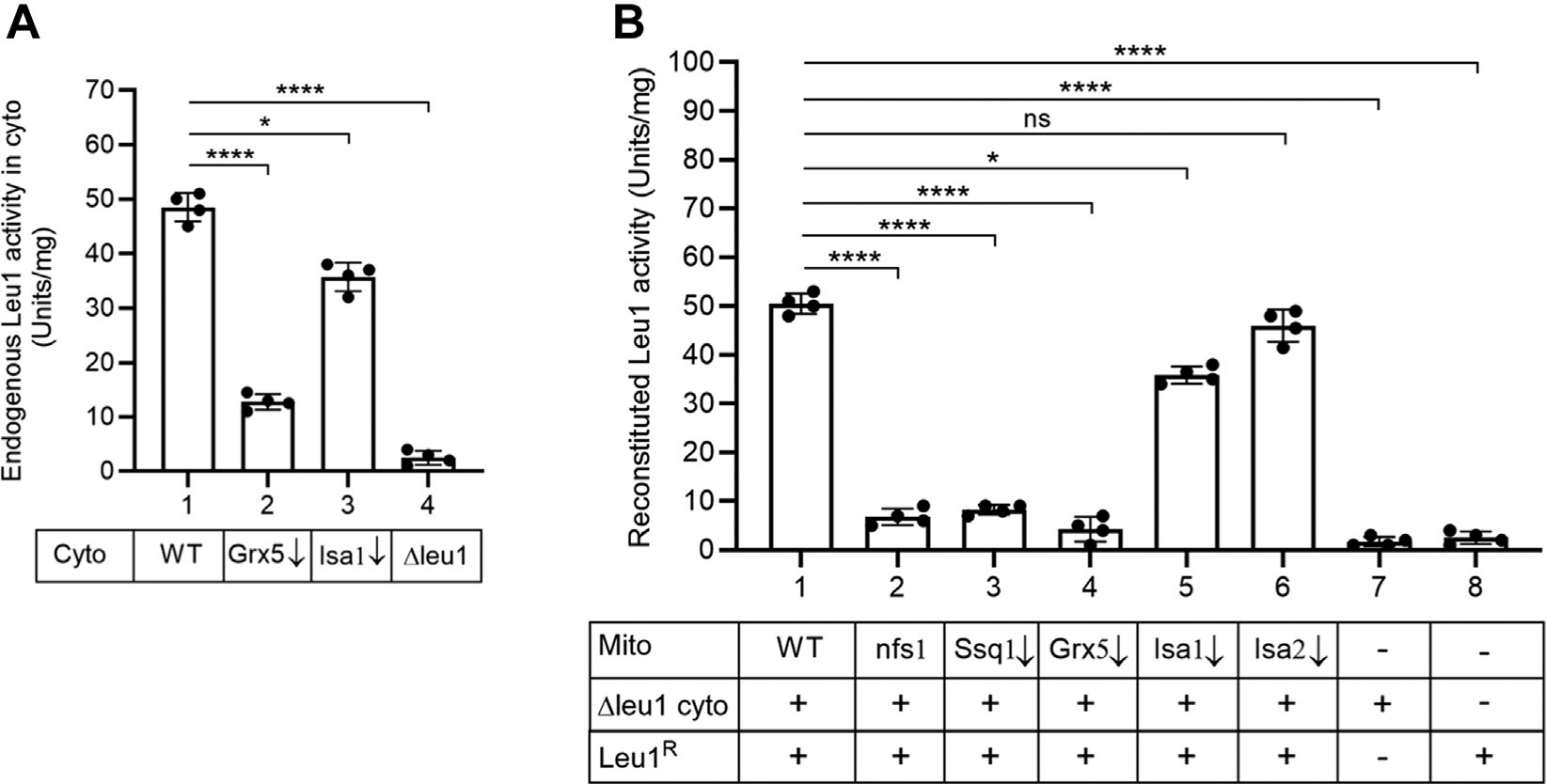
**Mitochondria lacking Grx5 cannot promote Leu1 [4Fe-4S] cluster assembly in cytoplasm.***A*, cytoplasm was isolated from WT, Gal-Grx5 repressed (Grx5↓), Gal-Isa1 repressed (Isa1↓), and *LEU1* gene deleted (Δleu1) strains, and endogenous Leu1 isopropylmalate isomerase activity was measured in samples containing 200 μg of proteins (43, 44). Data shown are the means ± SD (n = 4). *B*, isolated mitochondria (WT, nfs1, Ssq1↓, Grx5↓, Isa1↓ or Isa2↓; 200 μg of proteins) were mixed with isolated Δleu1 cytoplasm (200 μg of proteins) and apo-Leu1^R^ protein (2 μg). The Δleu1 cytoplasm alone or apo-Leu1^R^ protein alone served as background controls. All reaction mixtures were supplemented with unlabeled cysteine (10 μM), nucleotides (4 mM ATP, 1 mM GTP, 2 mM NADH), and iron (10 μM) and incubated at 30 °C for 30 min. After removal of mitochondria by centrifugation, the resulting cytoplasm/supernatant fractions were assayed for reconstituted Leu1^R^ isopropylmalate isomerase activity as in (*A*) above. Mutant nfs1, hypomorphic allele of Nfs1; Ssq1↓, Gal-Ssq1 repressed (42, 57). Data shown are the means ± SD (n = 4). Leu1^R^, recombinant Leu1; Grx5, glutaredoxin 5.

To further define the role of Grx5 in cytoplasmic Fe-S cluster assembly, a complementary but different assay was utilized. This time we used cytoplasmic isopropylmalate (IPM) isomerase (Leu1) as a target substrate. In *S. cerevisiae*, Leu1 in the cytoplasm isomerizes α-IPM to β-IPM, a critical step for leucine biosynthesis (55). The Leu1 enzyme activity is strictly dependent on proper assembly of its [4Fe-4S] cluster and thus serves as an indicator of cytoplasmic Fe-S cluster assembly status (37, 43, 56). An enriched cytoplasmic fraction (Fig. S1) was isolated from WT, Grx5-depleted (Grx5↓), Isa1-depleted (Isa1↓), and Δleu1 cells. As expected, robust activity of endogenous Leu1 was detected in WT cytoplasm in a spectrophotometric assay (Fig. 5*A*, bar 1). Interestingly, the cytoplasm isolated from Grx5↓ cells exhibited greatly reduced activity, with ∼20% of WT cytoplasmic activity (Fig. 5*A*, bar 2). In contrast, Isa1 depletion had a marginal effect and most (∼70%) of the Leu1 activity in the cytoplasm was preserved (Fig. 5*A*, bar 3). The Δleu1 strain lacks the gene for IPM isomerase and the isolated cytoplasm exhibited little, if any, Leu1 background activity (Fig. 5*A*, bar 4). This deletion strain, however, appears fully normal with respect to ISC and CIA activities (43). This allowed us to use apo-Leu1 as a substrate for cytoplasmic Fe-S cluster assembly in assays involving mitochondria from various sources mixed with Δleu1 cytoplasm as follows.

Briefly, the Leu1 protein with a C-terminal His_6_ tag was expressed in bacteria and purified (43). The purified recombinant protein, called recombinant Leu1 (Leu1^R^), was most likely in apo-form, as judged by its little, if any, enzyme activity (Fig. 5*B*, bar 8). The Δleu1 cytoplasm was mixed with mitochondria isolated from various strains and the samples were incubated with the Leu1^R^ protein under conditions that stimulate Fe-S cluster assembly, that is, in the presence of cysteine, nucleotides (ATP, GTP, and NADH), and iron. Mitochondria were removed by centrifugation and the cytoplasm/supernatant fractions were assayed for reconstituted Leu1 activity. In the presence of WT mitochondria, the Leu1^R^ apoprotein was found to be greatly activated, now exhibiting robust enzyme activity (Fig. 5*B*, compare bar 1 with bars 7 and 8). No such Leu1^R^ activation was observed with Grx5↓ mitochondria (Fig. 5*B*, bar 4) or mitochondria lacking upstream ISC components such as Nfs1 or Ssq1 (Fig. 5*B*, bars 2 and 3, respectively). In contrast, mitochondria lacking Grx5 downstream components (Isa1↓ or Isa2↓) were almost as effective as WT mitochondria in promoting activation of Leu1^R^ enzyme activity (Fig. 5*B*, bars 5 and 6). These results suggest that WT mitochondria generated the (Fe-S)_int_ and exported it to the cytoplasm. The exported intermediate was then utilized for the cytoplasmic synthesis of a new [4Fe-4S] cluster that was subsequently inserted into apo-Leu1^R^, generating the active holo enzyme. Grx5 and the other upstream core ISC components were necessary for synthesis of the (Fe-S)_int_, and mitochondria lacking any of these components failed to promote cytoplasmic Leu1^R^ activation. In contrast, Grx5 downstream components (*e.g.*, the ISA complex) were unnecessary for (Fe-S)_int_ production and/or export, and depletion of Isa1 or Isa2 did not interfere with mitochondrial ability to promote Leu1^R^ activation. This notion was further validated as described below.

### Grx5, but not ISA components, required for reactivation of defective cytoplasm in an nfs1 mutant

**Figure 6:**
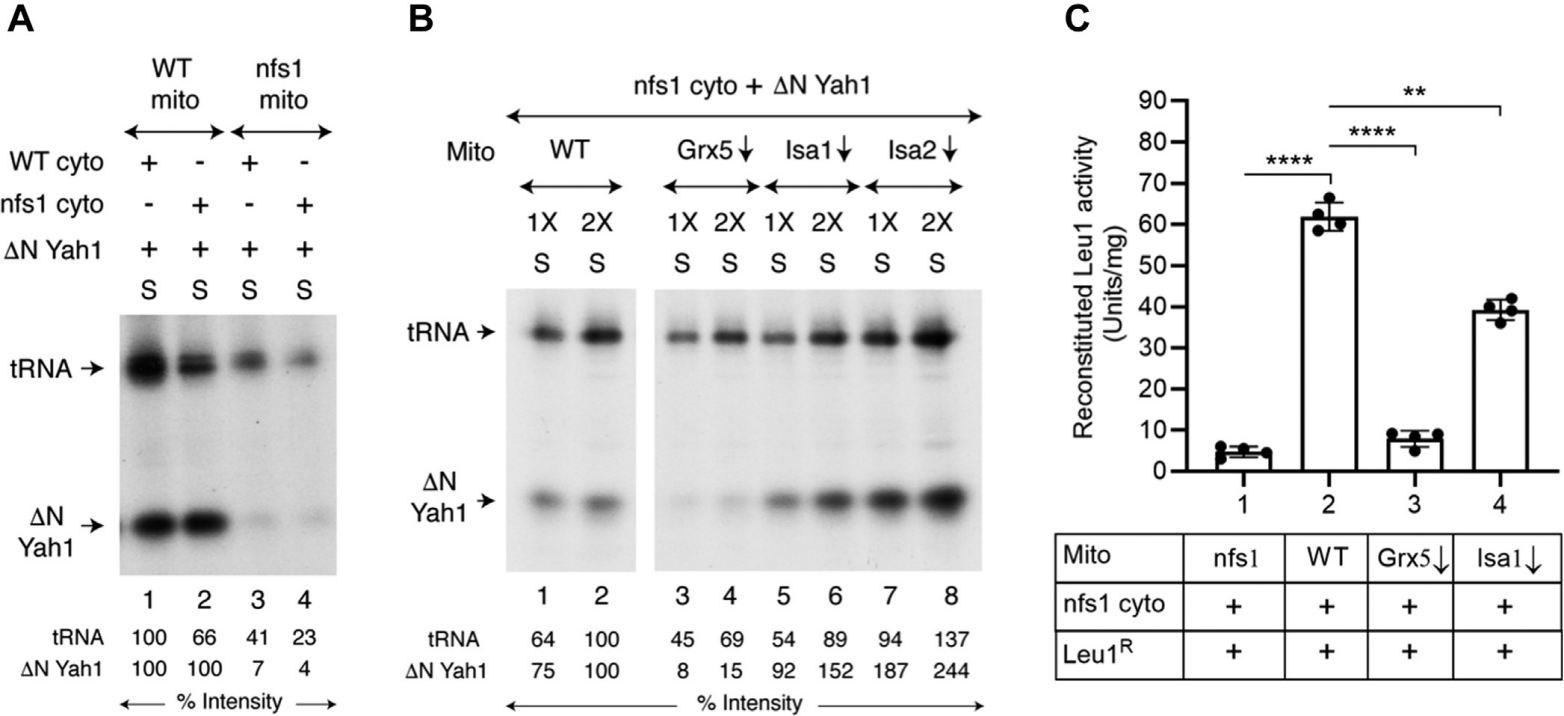
**Mitochondria lacking Grx5 cannot activate defective cytoplasm in the nfs1 mutant required for reconstitution of cytoplasmic Fe-S cluster assembly.***A*, mitochondria and cytoplasm were isolated from WT and nfs1 strains and as indicated, they were mixed in different combinations. Samples were supplemented with [^35^S]cysteine (10 μCi), nucleotides (4 mM ATP, 1 mM GTP, 2 mM NADH), iron (10 μM), and apo-ΔNYah1 protein (1 μg) and incubated at 30 °C for 30 min. Mitochondria were removed from assay mixtures by centrifugation, and the resulting cytoplasm/supernatant (“S”) fractions were analyzed by native PAGE, followed by autoradiography. *B*, mitochondria (WT, Grx5↓, Isa1↓, or Isa2↓; 200 μg of proteins) were mixed with nfs1 cytoplasm (200 μg of proteins) and apo-ΔNYah1 protein (1 μg). Samples were supplemented with [^35^S]cysteine, nucleotides, and iron, and then incubated at 30 °C for 30 min. After centrifugation, the cytoplasm/supernatant (“S”) fractions were analyzed as in (*A*) above. The *vertical dividing line* (between lanes 2 and 3) indicates removal of an unnecessary sample from the same autoradiograph. *C*, as indicated, mitochondria (nfs1, WT, Grx5↓, or Isa1↓; 200 μg of proteins) were mixed with cytoplasm isolated from the nfs1 strain (200 μg of proteins) and apo-Leu1^R^ protein (2 μg). Reaction mixtures were supplemented with unlabeled cysteine (10 μM), nucleotides (4 mM ATP, 1 mM GTP, 2 mM NADH), and iron (10 μM) and incubated at 30 °C for 30 min. Mitochondria were removed by centrifugation, and the resulting cytoplasm/supernatant fractions were assayed for reconstituted Leu1^R^ isopropylmalate isomerase activity as in Figure 5*B*. Data shown are the means ± SD for four biological replicates. ΔNYah1, N-terminal 60 amino acids including the mitochondrial targeting signal removed from the Yah1 precursor protein (pYah1); Leu1^R^, recombinant Leu1; Grx5, glutaredoxin 5; Fe-S, iron-sulfur.

The hypomorphic mutant nfs1-14 (called nfs1 for brevity) carries a missense *NFS1* allele (I191S) (57, 58). Mitochondria and cytoplasm were isolated from this nfs1 mutant, and mitochondria-cytoplasm mixing assays were performed in different combinations. The cytoplasmic ^35^S-tRNA signals served as internal controls (Fig. 6*A*, lanes 1–4). The nfs1 mitochondria are highly deficient in cysteine desulfurase activity and almost completely failed to promote Fe-^35^S cluster assembly of ΔNYah1 in cytoplasm derived from nfs1 cells (called “nfs1 cyto”) (Fig. 6*A*, lane 4). Mixing of WT mitochondria and nfs1 cytoplasm, however, exhibited efficient Fe-^35^S cluster loading of cytoplasmic ΔNYah1 (Fig. 6*A*, lane 2). These results were intriguing, because the CIA machinery in the cytoplasm contains several Fe-S proteins, including Dre2 of the electron transfer complex, Cfd1 and Nbp35 of the scaffold complex, and Nar1 of the targeting complex (17). One would expect these CIA components to be inactive or “latent” in the nfs1 cytoplasm and consequently, the nfs1 cytoplasm would be defective for Fe-S cluster assembly. Yet, addition of WT mitochondria (but not nfs1 mitochondria) restored efficient Fe-^35^S loading of added ΔNYah1 (Fig. 6*A*, compare lanes 2 and 4). A possibility is that WT mitochondria (with complete and productive ISC machinery) synthesized the (Fe-S)_int_ and subsequently exported it to the nfs1 cytoplasm. Once exported, the intermediate was utilized by the nfs1 cytoplasm, incorporating it into new Fe-S clusters or triggering a signal for the biosynthetic activity. Deficient Fe-S clusters of the CIA were replenished and/or latent CIA components were reactivated, and the cytoplasm regained the capacity for Fe-S cluster assembly. This hypothesis was further tested to distinguish the requirements for Grx5 and downstream ISA components in the process of reactivation of defective nfs1 cytoplasm as follows.

Briefly, increasing concentrations of different mitochondria were added to a mixture containing fixed concentrations of nfs1 cytoplasm and apo-ΔNYah1 protein. Assay mixtures were supplemented with [^35^S]cysteine, nucleotides, and iron. After incubation, samples were centrifuged, and the cytoplasm/supernatant (“S”) fractions were analyzed by native PAGE, followed by autoradiography. As shown in Fig. 6*B*, the Fe-^35^S labeling of ΔNYah1 in nfs1 cytoplasm was observed with WT (lanes 1 and 2), Isa1↓ (lanes 5 and 6), and Isa2↓ (lanes 7 and 8) mitochondria. Practically no such radiolabeling of ΔNYah1was observed with Grx5↓ mitochondria added to nfs1 cytoplasm (Fig. 6*B*, lanes 3 and 4). Most likely, mitochondria lacking Grx5 failed to generate the (Fe-S)_int_ that must be exported to the nfs1 cytoplasm for activation of “latent” CIA component(s) and/or subsequent cytoplasmic Fe-S cluster assembly. Isa1↓ or Isa2↓ mitochondria likely contained active Grx5 protein and thus behaved like WT mitochondria in terms of organellar synthesis of the (Fe-S)_int_ that is needed for promoting the downstream activation/assembly processes in the cytoplasm. Interestingly, the radiolabeled signals for ΔNYah1 appeared significantly more with Isa1↓ or Isa2↓ mitochondria compared to those with WT mitochondria (Fig. 6*B*). Unlike in the case of WT mitochondria, Fe-^35^S cluster trafficking from Grx5 to other recipients such as Aco1 was blocked in Isa1↓ or Isa2↓ mitochondria (*e.g.*, Fig. 1, *A*–*C*, right panels) and therefore, more of added [^35^S]cysteine was likely available/utilized for (Fe-^35^S)_int_ production within mitochondria, ultimately leading to more efficient radiolabeling of cytoplasmic ΔNYah1 (Fig. 6*B*).

We then tested reconstitution of Leu1^R^ [4Fe-4S] cluster assembly as judged by activation of its enzyme activity in similar settings as follows. Briefly, mitochondria (WT, nfs1, Grx5↓, or Isa1↓) were incubated with a mixture of nfs1 cytoplasm and apo-Leu1^R^, in the presence of unlabeled cysteine, nucleotides, and iron. Samples were centrifuged to remove mitochondria, and the resulting cytoplasm/supernatant fractions were assayed for reconstituted Leu1 activity. WT and nfs1 mitochondria served as positive and negative controls, respectively, for Leu1^R^ enzyme activation. Grx5↓ mitochondria behaved like nfs1 mitochondria and failed to promote Leu1^R^ enzyme activation over the background level (Fig. 6*C*, compare bars 1 and 3). In contrast, Isa1↓ mitochondria were almost (∼70%) as effective as WT mitochondria (Fig. 6*C*, compare bars 2 and 4). These results further substantiate the notion that Grx5 in mitochondria is required for the production of (Fe-S)_int_, which when exported to the cytoplasm is utilized for cytoplasmic Fe-S cluster biogenesis/homeostasis.

### Restoring (Fe-S)_int_ production in Grx5↓ mitochondria by importing purified Grx5

**Figure 7:**
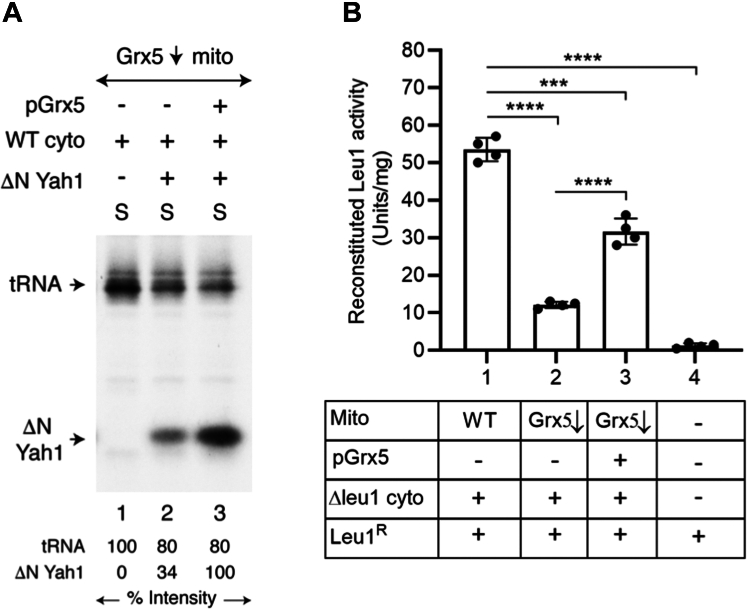
**Correcting inability of Grx5↓ mitochondria to promote cytoplasmic Fe-S cluster assembly by importing purified Grx5 protein.***A*, mitochondria lacking endogenous Grx5 (Grx5↓; 200 μg of proteins) were mixed with WT cytoplasm (200 μg of proteins), and assay mixtures were supplemented with [^35^S]cysteine, nucleotides, and iron. As indicated, the Grx5 precursor protein (pGrx5; 1 μg) and/or ΔNYah1 protein (1 μg) were added and samples were incubated at 30 °C for 30 min. Mitochondria were removed by centrifugation and the resulting cytoplasm/supernatant (“S”) fractions were analyzed by native PAGE, followed by autoradiography. *B*, mitochondria (WT or Grx5↓; 200 μg of proteins) were mixed with Δleu1 cytoplasm (200 μg of proteins) and apo-Leu1^R^ protein (2 μg). The pGrx5 protein (1 μg) was included as indicated. Apo-Leu1^R^ alone served as the background control. All reaction mixtures were supplemented with unlabeled cysteine (10 μM), nucleotides (4 mM ATP, 1 mM GTP, 2 mM NADH), and iron (10 μM) and incubated at 30 °C for 30 min. After removal of mitochondria by centrifugation, and the cytoplasm/supernatant fractions were assayed for reconstituted Leu1^R^ isopropylmalate isomerase activity as in Figure 5*B*. Data shown are the means ± SD (n = 4). ΔNYah1, N-terminal 60 amino acids including the mitochondrial targeting signal removed from the Yah1 precursor protein (pYah1); Leu1^R^, recombinant Leu1; Grx5, glutaredoxin 5; Fe-S, iron-sulfur.

Isolated Grx5↓ mitochondria failed to synthesize/assemble new Fe-S clusters for aconitase (Aco1), and this defect was greatly corrected by importing purified Grx5 (Fig. 2, *A*–*C*). The Grx5↓ mitochondria by themselves also failed to promote cytoplasmic Fe-S cluster assembly (Figs. 4*A* and 5*B*). Can this cytoplasmic defect be also corrected by newly imported Grx5 protein? To test this possibility, a mixture of Grx5↓ mitochondria and WT cytoplasm was incubated with [^35^S]cysteine, nucleotides, and iron, with or without added ΔNYah1. The purified pGrx5 was included as indicated (Fig. 7*A*). After incubation, reaction mixtures were centrifuged to remove mitochondria, and the resulting cytoplasm/supernatant (“S”) fractions were analyzed by native PAGE, followed by autoradiography. Addition of pGrx5 to Grx5↓ mitochondria greatly stimulated ^35^S-labeling of cytoplasmic ΔNYah1 (Fig. 7*A*, compare lanes 2 and 3, bottom bands). Under these assay conditions, pGrx5 was imported into Grx5↓ mitochondria and restored efficient (Fe-^35^S)_int_ synthesis within mitochondria. The intermediate was exported from mitochondria to the cytoplasm and greatly stimulated Fe-^35^S cluster assembly/loading of cytoplasmic ΔNYah1. Notably, cytoplasmic ^35^S-tRNA thiolation was practically unaffected by pGrx5 imported into Grx5↓ mitochondria (Fig. 7*A*, compare lanes 2 and 3, top bands). Cytoplasmic tRNA thiolation occurred independent of Grx5 (also see Fig. 4*A*). The process requires a different mitochondria-synthesized S_int_, and S_int_ synthesis occurs independent of Grx5.

To further confirm the stimulatory role of imported Grx5 on cytoplasmic Fe-S cluster assembly, we performed similar assays, looking for reconstitution of Leu1^R^ enzyme activity. Briefly, a mixture of mitochondria (WT or Grx5↓) and Δleu1 cytoplasm was incubated with apo-Leu1^R^ in the presence of unlabeled cysteine, nucleotides, and iron. The pGrx5 was added as indicated (Fig. 7*B*). Samples were centrifuged and the resulting cytoplasm/supernatant fractions were evaluated for reconstituted [4Fe-4S] cluster assembly of Leu1 as judged by IPM isomerase activity. Imported Grx5 enhanced the ability of Grx5↓ mitochondria to promote cytoplasmic Leu1 enzyme activation by ∼3-fold, approaching to ∼60% of Leu1^R^ activation observed with WT mitochondria (Fig. 7*B*, compare bars 1–3). Thus, Grx5 imported into Grx5↓ mitochondria can greatly restore (Fe-S)_int_ synthesis within mitochondria, which when exported is utilized by the cytoplasm for [2Fe-2S] cluster assembly of ΔNYah1 (Fig. 7*A*) or [4Fe-4S] cluster assembly of Leu1^R^ (Fig. 7*B*). In summary, Grx5 plays an essential role in overall Fe-S cluster trafficking for maintaining mitochondrial/cellular Fe-S protein homeostasis.

## Discussion

**Figure 8:**
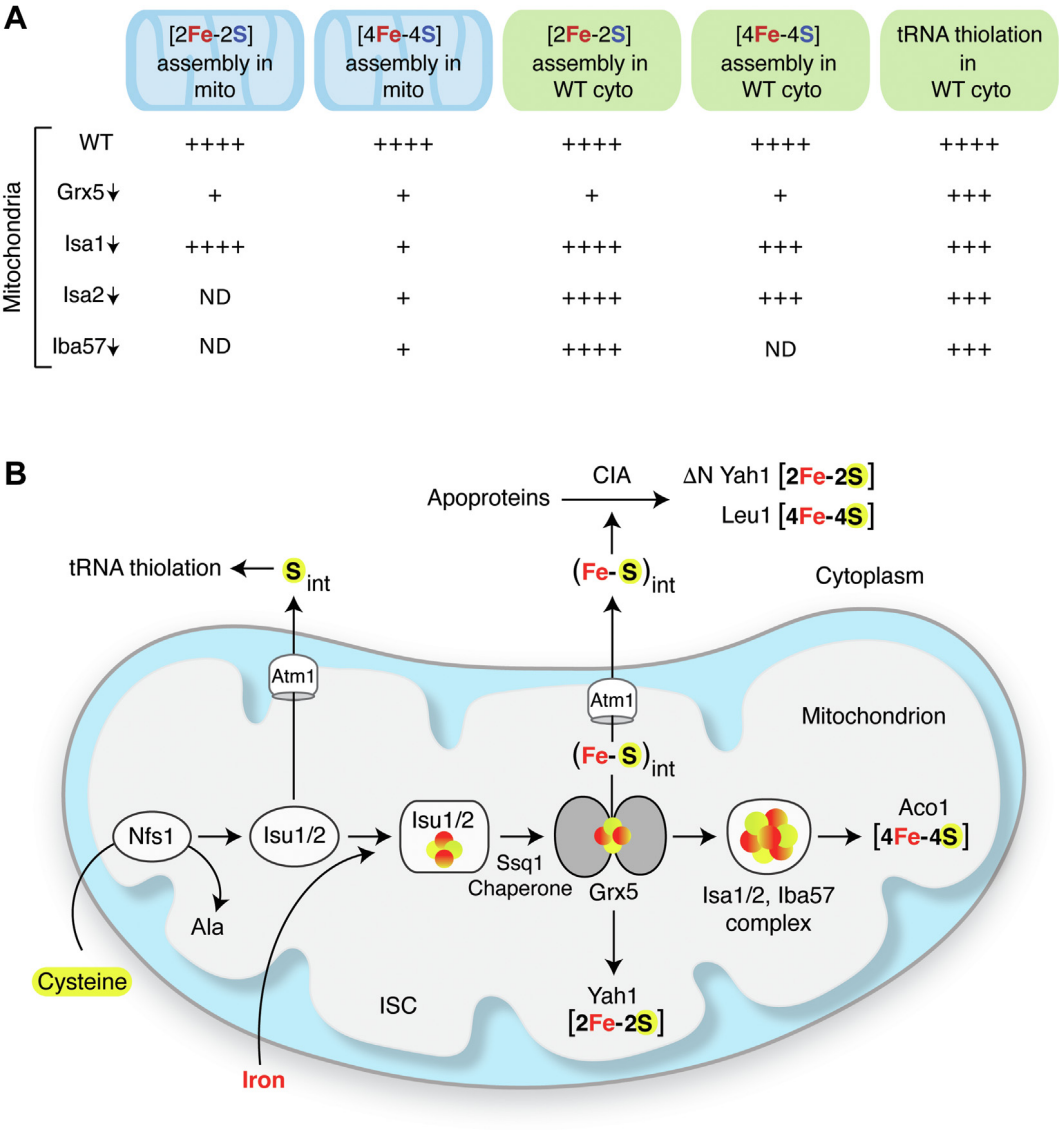
**Trafficking role of Grx5 in cellular Fe-S protein biogenesis.***A*, summary of the results presented here. For all biosynthetic processes tested, the efficiency of WT mitochondria was arbitrarily considered 100% as indicated by four pluses (“++++”). ND, not determined. *B*, a model for Grx5 acting at the focal point of “*three-way*” Fe-S cluster trafficking in mitochondria. In the conventional mitochondrial pathway, the ISC machinery synthesizes [2Fe-2S] and [4Fe-4S] clusters for organellar iron proteins (16, 20). The core components of the machinery are also involved in producing two different intermediates: S_int_ for cytoplasmic tRNA thiolation and (Fe-S)_int_ for cytoplasmic Fe-S cluster assembly (42). The ISC pathway begins with the activity of a protein complex consisting of Nfs1 and other factors (not shown). The Nfs1 cysteine desulfurase abstracts sulfur from the amino acid cysteine to form a persulfide sulfur intermediate, which is then donated to the Isu1/2 scaffold. At this stage, this sulfur may leave the conventional ISC pathway and is utilized for S_int_ formation in a process that does not require downstream components such as the Ssq1 chaperone (42), Grx5 (Fig. 4*A*), or the ISA complex (Fig. 4, *B*–*D*). As needed, the S_int_ is exported to the cytoplasm where it is utilized for thiolation of tRNAs (42, 53, 54). In the context of Fe-S cluster assembly, however, the conventional ISC pathway continues with Isu1/2 forming a [2Fe-2S] cluster intermediate by combining the persulfide sulfur from Nfs1 and iron from an undetermined source. Ssq1 (together with other proteins; not shown) then promotes transfer of this intermediate to Grx5 (16, 20). At this stage, Grx5 controls “*three-way*” trafficking of Fe-S clusters/intermediates. First, the [2Fe-2S] cluster may be directly transferred from Grx5 to a recipient such as ferredoxin (Yah1), with no further requirement of any other downstream ISC components such as the ISA complex (Fig. 3). Second, Grx5 may transfer [2Fe-2S] cluster to the ISA complex, which generates a [4Fe-4S] cluster by reductive coupling for aconitase. The aconitase [4Fe-4S] cluster assembly is blocked in mitochondria lacking Grx5 or Isa1 (Fig. 1), but the biosynthetic process is efficiently restored by newly imported Grx5 or Isa1 into respective mutant mitochondria (Fig. 2, *A*–*D*). Finally, Grx5 also plays a vital role for (Fe-S)_int_ synthesis. Here, the pathway branches again. The (Fe-S)_int_ moves from Grx5 to the Atm1 transporter and is exported to the cytoplasm. Once exported, (Fe-S)_int_ is utilized by the CIA machinery, generating cytoplasmic Fe-S clusters (42). Grx5-depleted mitochondria cannot promote [2Fe-2S] or [4Fe-4S] cluster assembly (Figs. 4 and 5, respectively) but can efficiently do so with the help of newly imported Grx5 (Fig. 7). Notably, both iron and sulfur for cytoplasmic Fe-S cluster assembly likely originate from the mitochondria (42), revealing an essential and direct role of Grx5 in (Fe-S)_int_ synthesis and trafficking. CIA, cytoplasmic iron-sulfur protein assembly machinery; Fe-S, iron-sulfur; (Fe-S)_int_, (Fe-S) intermediate; Grx5, glutaredoxin 5; IPM, isopropylmalate; ISC, mitochondrial iron-sulfur cluster.

In eukaryotes, the biosynthesis of Fe-S cluster cofactors requires a mitochondrial iron-sulfur cluster (ISC) machinery and a cytoplasmic iron-sulfur protein assembly (CIA) machinery. Whereas these two machineries are confined to distinct biochemical and metabolic environments, they are functionally linked for maintaining overall cellular Fe-S protein homeostasis (16, 20). The monothiol glutaredoxin Grx5 is a component of the mitochondrial ISC machinery and is conserved from yeast to zebra fish to humans. Its deficiency *in vivo* results in Fe-S protein deficiencies throughout the cell. In humans, mutated alleles of Grx5 have been linked to lethal human diseases such as sideroblastic anemia or spasticity with hyperglycinemia (5, 10, 11, 12, 39). Here, we have investigated the role of Grx5 step by step in mitochondrial/cellular Fe-S protein biogenesis in yeast, starting from steady-state *in vivo* Fe-S protein activities to new synthesis, assembly, and/or reconstitution of Fe-S clusters in isolated mitochondria and cytoplasm. Results from this comprehensive study are summarized in Figure 8*A*, providing evidence for Grx5 functioning as a central hub for Fe-S cluster trafficking inside and outside of mitochondria (Fig. 8*B*).

The ISC machinery consists of as many as 18 proteins in the mitochondrial matrix (20). Most of these proteins, including Grx5, are encoded by the nuclear DNA, synthesized on cytoplasmic ribosomes, and imported into the mitochondrial matrix via N-terminal mitochondrial targeting sequences. Notably, the mitochondrial ISC machinery lies upstream of the CIA machinery in the cytoplasm (42, 56) (see below), making the overall process of cellular Fe-S protein homeostasis very complex. The phenotypes associated with Grx5 deficiency/depletion have thus far been mostly studied in whole cells of different species (5, 10, 11, 12, 39). In intact cells, however, it is difficult to biochemically demarcate/separate processes occurring specifically in mitochondria from those occurring in the cytoplasm for cellular Fe-S protein biogenesis. Consequently, defining the mechanistic steps involved in Fe-S cluster trafficking/assembly continues to be challenging based solely on *in vivo* whole-cell experiments. Ideally, *in vivo* results should be validated with complementary biochemical assays involving isolated mitochondria and/or cytoplasm. Such assays have thus far not been described for Grx5 or other downstream components of the ISC machinery (*e.g.*, Isa1), and here we filled this gap.

Mitochondria lacking Grx5 or the immediate downstream ISA components (Isa1, Isa2, or Iba57) exhibited very little aconitase (Aco1) activity (Fig. 1, *A*–*D*, left panels; Fig. S2*A*). These mutant mitochondria, however, contained Aco1 protein comparable to the WT level (Fig. S3). This allowed us to test if these mitochondria are capable of synthesizing new [4Fe-4S] clusters that can be incorporated into endogenous Aco1. For this purpose, isolated mitochondria were incubated with [^35^S]cysteine under conditions that promote Fe-S cluster synthesis, that is, in the presence of added iron and nucleotides. As expected, WT mitochondria generated Fe-^35^S clusters, which were inserted into endogenous Aco1, making the protein radiolabeled. However, only little radiolabeled Aco1 was detected in mitochondria lacking Grx5, Isa1, Isa2, or Iba57 (Fig. 1, *A*–*D*, right panels, respectively), indicating an apparently similar defect in [4Fe-4^35^S] cluster biosynthesis.

Most importantly, bacterial expressed and purified Grx5 imported into mitochondria lacking endogenous Grx5 (*i.e.*, Gal-Grx5↓ mitochondria) efficiently restored Fe-^35^S cluster synthesis and loading of Aco1 with radiolabeled clusters (Fig. 2, *A*–*C*). Similarly, purified Isa1, imported into mitochondria lacking endogenous Isa1 (*i.e.*, Gal-Isa1↓ mitochondria), reestablished Aco1 loading almost to WT level (Fig. 2*D*). Thus, lack of Aco1 activity in these mutant mitochondria was most likely due to lack of [4Fe-4S] cluster synthesis/assembly *in vivo*. The defect was not due to secondary/nonspecific and/or irreversible effects on Fe-S cluster biosynthetic processes that might occur during *in vivo* depletion of Grx5 (or Isa1). Rather, the aconitase loading defect was specifically due to deficiency of Grx5 (or Isa1) as it was reversed by import of purified Grx5 (or Isa1). Grx5 itself requires an Fe-S cluster for function (35, 59). In Grx5↓ mitochondria, the newly imported Grx5 protein likely acquired a transient [2Fe-2S] cluster through actions of upstream components (*e.g.*, Nfs1 and its accessory proteins, Isu1/2 scaffold, Ssq1 chaperone *etc.*) and donated it to the ISA complex for further processing that is needed for aconitase [4Fe-4S] cluster loading. Such studies have never been done before with isolated mitochondria to define the role of Grx5 (or Isa1).

The [2Fe-2S] cluster of bacterial expressed and chemically reconstituted Grx5 appears to be intrinsically labile; it can be rapidly transferred to purified mitochondrial and/or cytoplasmic apoproteins *in vitro* (35, 60, 61), pointing to an Fe-S cluster carrier and/or donor function for the Grx5 protein (16, 20, 60). Such a transfer process, however, was achieved with a nonphysiological and strong chemical thiol reductant (*e.g.*, DTT), without requiring any ISC and/or CIA components. An elegant *in vitro* reconstitution assay without DTT has recently been developed using purified human proteins. In this assay, Fe-S cluster transfer from human GLRX5 to aconitase required not only the ISA complex but also the electron transfer chain NADPH-ferredoxin reductase-ferredoxin (35). In our assays with isolated mitochondria, Grx5 but not Isa1 was found to be necessary for [2Fe-2S] cluster synthesis/loading of imported yeast ferredoxin (Yah1) (Fig. 3). These data suggest an important role of Grx5 in [2Fe-2S] cluster trafficking to Yah1, Isa1, and perhaps other recipients in mitochondrial setting (Fig. 8*B*). Notably, the mitochondrial ferredoxin itself requires a [2Fe-2S] cluster for function (27, 29). Thus, there appears to be a chicken and egg conundrum here, since ferredoxin [2Fe-2S] is known to act upstream to generate a transient Fe-S cluster on Grx5 (16, 20). The details remain to be worked out.

An essential role of mitochondria in cytoplasmic Fe-S cluster biogenesis became apparent from a seminal *in vivo* experiment by Lill and co-workers (56). The experiment involved depletion of the mitochondrial Nfs1 cysteine desulfurase (or the Atm1 exporter) from yeast cells using a regulated promoter, which resulted in loss of cytoplasmic IPM isomerase (Leu1) activity due to lack of its Fe-S cluster. This finding led to the proposal that the mitochondrial ISC via Nfs1 produces an intermediate called X-S, because it may contain a sulfur atom. The X-S intermediate is then exported from mitochondria by the ABC transporter Atm1 in the mitochondrial inner membrane to the cytoplasm for cytoplasmic Fe-S cluster assembly (17, 56). More recently, we developed biochemical assays to directly test this mitochondria–cytoplasm interaction using mitochondria and cytoplasm isolated from defined yeast cells as described below (42, 43, 44, 54).

Mitochondria isolated from a WT strain contain a complete ISC machinery and are capable of synthesizing new Fe-S clusters (40, 48). In contrast, cytoplasm isolated from the same WT strain contains CIA components, but this cytoplasmic system is not autonomous and cannot make Fe-S clusters on its own (42). Also, isolated WT cytoplasm by itself cannot thiolate cytoplasmic tRNAs specific for lysine, glutamate, and glutamine, a process required for accurate protein synthesis (53, 54). This prompted us to develop mitochondria-cytoplasm mixing assays, demonstrating that a mixture of mitochondria and cytoplasm is required for both tRNA thiolation and Fe-S cluster assembly in the cytoplasm, as both make critical contributions (42, 43, 44, 54). Specifically, we found that the mitochondrial ISC synthesizes two distinct intermediates: S_int_ and (Fe-S)_int_. The intermediates are exported to the cytoplasm via the Atm1 transporter. Once exported, S_int_ is utilized for thiolation of the cytoplasmic tRNAs by a sulfur relay (Fig. 8*B*) (53, 54). On the other hand, the (Fe-S)_int_ exported from mitochondria is utilized by the CIA machinery; this intermediate likely provides both iron and sulfur species required for cytoplasmic Fe-S cluster assembly (Fig. 8*B*) (42). A major advantage of these mixing assays is that specific/direct contributions of mitochondria and cytoplasm isolated from different genetic backgrounds can be tested side by side rapidly and reproducibly. For example, mitochondria isolated from various ISC mutant strains can be combined with WT cytoplasm, thereby avoiding any secondary effects on the CIA components. Here, this unique assay system was further advanced by combining with mitochondrial protein import, and this allowed us to define the role of mitochondrial Grx5 in cytoplasmic processes with confidence as follows.

In a typical assay, a mixture of mitochondria (WT or Grx5↓) and WT cytoplasm was incubated with [^35^S]cysteine, nucleotides, and iron. Grx5↓ mitochondria were able to promote robust cytoplasmic ^35^S-tRNA signals as efficiently as observed with WT mitochondria (Fig. 4*A*). These results indicate that Grx5 is not required for S_int_ formation (Fig. 8*A*), consistent with our earlier finding that the Isu1/2 scaffold (Grx5 upstream component) constitutes a branch point for formation of this intermediate (Fig. 8*B*) (42). Importantly, however, Grx5 appears to serve at the critical branch point of Fe-S cluster trafficking, leading to (Fe-S)_int_ biosynthesis in mitochondria, followed by its export from the organelle to cytoplasm for cytosolic Fe-S cluster assembly (Fig. 8*B*). This notion is supported by several independent observations. 1) When supplemented with [^35^S]cysteine, nucleotides and iron, only WT mitochondria, but not Grx5↓ mitochondria, were able to synthesize (Fe-^35^S)_int_ and promoted [2Fe-2^35^S] cluster assembly of the ΔNYah1 protein in WT cytoplasm (Fig. 4*A*). 2) Similarly, when supplemented with unlabeled cysteine, nucleotides and iron, only WT but not Grx5↓ mitochondria promoted [4Fe-4S] cluster assembly on purified Leu1^R^ in Δleu1 cytoplasm, as judged by reconstitution of the Leu1 enzyme activity (Fig. 5*B*). 3) Most importantly, purified Grx5 precursor protein (pGrx5) imported into Grx5↓ mitochondria efficiently restored (Fe-S)_int_ biosynthesis and led to formation of radiolabeled ΔNYah1 in WT cytoplasm (Fig. 7*A*) or reconstituted Leu1 enzyme activity in Δleu1 cytoplasm (Fig. 7*B*). 4) Finally, components of the ISA complex were not needed for (Fe-S)_int_ biosynthesis/export (Figs. 4, *B*–*D* and 5*B*), placing Grx5 at the branch point and preceding ISA proteins (Fig. 8*B*).

In summary, Grx5 plays a vital role in overall cellular Fe-S protein homeostasis. It acts at the focal point of a “*three-way*” hub for Fe-S cluster trafficking in mitochondria (Fig. 8*B*). It is required for [2Fe-2S] cluster assembly of ferredoxin (Yah1) in mitochondria. Grx5 must also hand over a [2Fe-2S] cluster to the ISA complex for reductive coupling and formation of a [4Fe-4S] cluster for recipients such as aconitase (Aco1). In addition, Grx5 delivers the (Fe-S)_int_ to the mitochondrial export machinery including Atm1. Thus, Grx5 imported into Grx5↓ mitochondria was able to efficiently restore [4Fe-4S] cluster assembly of aconitase. The imported protein also greatly restored (Fe-S)_int_ synthesis within mitochondria, which when exported was utilized by the cytoplasm for [2Fe-2S] cluster assembly of ΔNYah1 or [4Fe-4S] cluster assembly of Leu1^R^. The precise mechanism by which Grx5 may regulate these various Fe-S cluster trafficking processes remains to be determined. The mitochondria-cytoplasm mixing assays combined with mitochondrial protein import as described here would be particularly useful toward achieving that goal.

## Experimental procedures

### Chemicals and reagents

Yeast nitrogen base without amino acids and individual amino acids were purchased from Thermo Fisher Scientific. Raffinose was from Gold Biotechnology. Galactose, sorbitol, ATP, GTP, NADH, NADP, ferrous ammonium sulfate, L-ascorbic acid, PMSF, Trasylol, phenazine methosulfate, *cis*-aconitic acid, isocitrate dehydrogenase, thiazolyl blue tetrazolium bromide, 3-isopropyl malic acid, valinomycin, carbonyl cyanide m-chlorophenyl hydrazone, and catalase were purchased from Sigma. [^35^S]cysteine (1000 Ci/mmol) and EXPRESS^35^S (1000 Ci/mmol) were purchased from Revvity Health Science Inc. Horseradish peroxidase–linked secondary antibodies (donkey anti-rabbit IgG and goat anti-mouse IgG) were purchased from GE Healthcare Life Sciences and Thermo Fisher Scientific, respectively.

### Yeast strains and culture conditions

Yeast strains used in this study are listed in Table S1. Four new strains were generated for this study: Gal-Grx5, Gal-Isa1, Gal-Isa2, and Gal-Iba57. For this purpose, the parental strain BY4741 was subjected to *GAL1* promoter swap at the respective gene locus, using the method described by Longtine *et al.* (45). Synthetic complete medium containing raffinose, with or without dextrose or galactose, were made as described (62). For isolation of mitochondria or cytoplasm (see below), the WT, nfs1-14 (also called nfs1), Gal-Ssq1, Gal-Grx5, Gal-Isa1, Gal-Isa2, Gal-Iba57, Δnfu1, Δbol3, or Δleu1 cells were grown in synthetic complete containing 2% raffinose, 0.5% dextrose medium at 30 °C for 16 to 70 h to an A_600_ ∼ 1 to 1.2.

### Mitochondrial and cytoplasmic preparations

Cells were harvested, and mitochondria and cytoplasm were isolated as described (43). Briefly, cells were treated with Zymolyase 100T, and spheroplasts thus generated were resuspended in ice-cold buffer A (20 mM Hepes/KOH, pH 7.5, 0.6 M sorbitol, 0.1% bovine serum albumin, 1 mM PMSF, and 10 U/ml Trasylol; ∼3.5 ml per gram of starting cells). After homogenization in a Dounce homogenizer on ice, samples were diluted with buffer A, and centrifuged at 1500*g* for 5 min at 4 °C. The supernatant was saved, and the pellet was rehomogenized, diluted, and centrifuged under the same conditions. The supernatant fractions were combined and centrifuged again at 1500*g* for 5 min at 4 °C. The supernatant obtained at this stage was centrifuged at 10,000*g* for 10 min at 4 °C to isolate enriched mitochondrial pellet. This mitochondrial fraction was resuspended and stored in isotonic Hepes-sorbitol (HS) buffer (20 mM Hepes/KOH, pH 7.5, containing 0.6 M sorbitol) with 0.1 mg/ml bovine serum albumin, 10 U/ml Trasylol, and 10% dimethyl sulfoxide in aliquots at −80 °C. As needed, frozen mitochondria were thawed, diluted with ice-cold HS buffer, and centrifuged at 10,000*g* for 2 min at 4 °C to remove dimethyl sulfoxide. The mitochondrial pellet was resuspended in HS buffer for further assays.

To isolate cytoplasm, spheroplasts were homogenized in ice-cold buffer A (∼1 ml per gram of starting cells) and centrifuged at 1500*g* for 5 min at 4 °C. The supernatant was centrifuged again at 10,000*g* for 10 min at 4 °C, removing mitochondria in the pellet. The postmitochondrial supernatant was finally centrifuged at 153,000*g* for 45 min at 4 °C, and the resulting supernatant/cytoplasmic fraction was stored in aliquots at −80 °C (43). As expected, mitochondrial marker proteins (Aco1 and Put2) and Aco1 activity were detected in the mitochondrial preparation but not in the cytoplasmic preparation. Conversely, cytoplasmic marker proteins (Dre2 and Pgk1) and Leu1 activity were specifically detected in the cytoplasmic preparation but not in the mitochondrial preparation (Fig. S1).

### Bacterial expression and purification of proteins

A) Mitochondrial precursor proteins pGrx5, pIsa1, and pYah1: These are full-length proteins with the corresponding N-terminal mitochondrial targeting signal intact. These precursor proteins with a C-terminal His_6_ tag were individually expressed in BL21 (DE3) cells carrying the plasmid pET21b with respective ORFs essentially as described (40). Briefly, cells were grown at 37 °C in Luria-Bertani (LB) media containing 100 μg/ml ampicillin to A_600_ of ∼0.8. Following addition of 1 mM IPTG, protein expression was continued for 20 h at the same temperature. Under these conditions, the precursor proteins (pGrx5, pIsa1, or pYah1) were found to be sequestered in inclusion bodies. The proteins were solubilized with 8 M urea in 50 mM Tris/HCl, pH 8.0, and centrifuged at 250,000*g* for 20 min at 20 °C to remove insoluble material. The supernatant fractions were highly enriched in the protein of interest and stored at −80 °C until further use. For some experiments, radiolabeled precursor proteins were desired and hence protein induction/expression was carried out in M9 media containing 100 μg/ml ampicillin, 10 μCi/ml EXPRESS^35^S, and IPTG at 37 °C for 3 h. Inclusion bodies containing ^35^S-labeled precursor proteins were solubilized with 8 M urea and processed as described above for the unlabeled proteins (63, 64).
B) Cytoplasmic apoprotein substrates ΔNYah1 and Leu1^R^: The N-terminal 60 amino acids including the mitochondrial targeting signal were removed from the pYah1, generating ΔNYah1 (42). BL21 (DE3) cells carrying the plasmid pET21b/ΔNYah1-His_6_ were grown in LB media containing 100 μg/ml ampicillin and 0.5 mM IPTG for 20 h at 20 °C. Under these conditions, the expressed protein was found mostly in soluble form, and it was purified by Ni-NTA affinity chromatography and stored frozen in aliquots at −80 °C. As needed, the purified protein was thawed, treated with 0.2 N HCl, and then neutralized with 1 M Tris/HCl, pH 8.0 (42). The apo-ΔNYah1 thus generated was used as a substrate for cytoplasmic Fe-S cluster assembly. Likewise, the cytoplasmic Leu1 protein with a C-terminal His_6_ tag was expressed in *Escherichia coli* and purified (43). Briefly, BL21 (DE3) cells harboring the plasmids pET21b/Leu1-His_6_ and pBB541-groESL were cultured in LB medium containing ampicillin (100 μg/ml) and spectinomycin (50 μg/ml). Protein expression was induced with 0.5 mM IPTG for 20 h at 16 °C. The Leu1-His_6_ protein was found in the soluble fraction, and it was purified using Ni-NTA affinity chromatography. The purified recombinant Leu1, called Leu1^R^, is predominantly in its apo-form and exhibits minimal IPM isomerase activity (43).

### Fe-S cluster assembly in isolated mitochondria

A standard reaction mixture contained isolated mitochondria (200 μg of proteins), [^35^S]cysteine (10 μCi), ATP (4 mM), GTP (1 mM), NADH (2 mM), ferrous ascorbate (10 μM), KOAc (40 mM), and Mg(OAc)_2_ (10 mM) in HS buffer (100 μl). Following incubation at 30 °C for 10 to 30 min, the reaction mixtures were diluted with 1 ml of HS buffer and centrifuged at 15,000*g* for 10 min at 4 °C. The resulting mitochondrial pellet (“P”) was resuspended in 40 μl of 50 mM Tris/HCl, pH 8.0 containing 1 mM PMSF, and the membranes were disrupted by freeze/thaw and bath sonication at 4 °C as described (40, 48). The samples were then centrifuged at 15,000*g* for 30 min at 4 °C, and the supernatant containing soluble matrix proteins was analyzed by native PAGE under reducing conditions, followed by autoradiography. Samples were evaluated for radiolabeling of endogenous aconitase (Aco1) due to insertion of newly made Fe-^35^S clusters. Similar assays were done with the ferredoxin precursor protein (pYah1) added to assay mixtures. Under these conditions, pYah1 was imported into mitochondria, the mitochondrial targeting signal was removed, and the mature ferredoxin thus generated served as a substrate for [2Fe-2^35^S] cluster assembly within the organelle. In some assays, pGrx5 or pIsa1 was included to determine effects of their import on mitochondrial Fe-^35^S cluster/intermediate biosynthesis. These variations are included in Figure legends as needed.

### Mitochondria-cytoplasm mixing assays for cytoplasmic Fe-S cluster assembly

A) Fe-^35^S radiolabeling of cytoplasmic ΔNYah1: Assays were performed with mitochondria and cytoplasm isolated from various yeast strains. A typical reaction mixture contained mitochondria (100–200 μg of proteins), cytoplasm (200 μg of proteins), apo-ΔNYah1 protein (1 μg), [^35^S]cysteine (10 μCi), ATP (4 mM), GTP (1 mM), NADH (2 mM), ferrous ascorbate (10 μM), KOAc (40 mM), and Mg(OAc)_2_ (10 mM) in HS buffer (100 μl). After incubation at 30 °C for 10 to 30 min, samples were centrifuged at 15,000*g* for 10 min at 4 °C. Proteins (and tRNAs) in the resulting cytoplasm/supernatant (“S”) fractions were precipitated with 67% ammonium sulfate on ice for 1 to 2 h. After centrifugation at 15,000*g* for 45 min at 4 °C, the protein and tRNA-containing pellets were dissolved in 50 mM Tris/HCl, pH 8.0 containing 1 mM PMSF and 10 mM DTT. Samples were analyzed by native PAGE followed by autoradiography, looking for radiolabeling of proteins with newly made Fe-^35^S clusters and radiolabeling of tRNAs resulting from ^35^S-thiolation (42, 54). Radiolabeled protein and tRNA bands were quantified by densitometric analysis of autoradiographs using the NIH ImageJ software (https://imagej.net). In most cases, data as presented are internally controlled. The reproducibility of various assays was confirmed with biological replicates from different batches of isolated mitochondria and/or cytoplasm.
B) Cytoplasmic Leu1^R^ enzyme activation: A typical assay mixture (100 μl) contained isolated mitochondria (200 μg of proteins), cytoplasm isolated from Δleu1 cells (200 μg of proteins), purified apo-Leu1^R^ protein (2 μg), unlabeled cysteine (10 μM), nucleotides (4 mM ATP, 1 mM GTP, 2 mM NADH), ferrous ascorbate (10 μM), KOAc (40 mM), and Mg(OAc)_2_ (10 mM) in 20 mM Tris–HCl buffer, pH 7.5 with 0.6 M sorbitol. Samples were incubated at 30 °C for 30 min and then centrifuged at 14,000*g* for 10 min at 4 °C. The resulting cytoplasm/supernatant fractions were assayed for reconstituted IPM isomerase activity (43). The enzyme assay detects formation of 2-IPM from 3-IPM for 15 min at 25 °C at 235 nm in a BioTek Synergy HTX spectrophotometer (37, 44, 65). One unit of activity corresponds to 18 nmole product formed/hour, and results are expressed in Units/mg of cytoplasmic proteins. Data were analyzed using GraphPad Prism, version 10 (https://www.graphpad.com/features). Comparisons between two groups were performed using unpaired Student’s *t* test. *P* values on all bar graphs are as follows: ∗*p* < 0.05, ∗∗*p* < 0.01, ∗∗∗*p* < 0.001, and ∗∗∗∗*p* < 0.0001.

### Miscellaneous

Aconitase activity was assessed using an in-gel assay as described (44, 54). Bands displaying aconitase activity appeared purple/blue. These bands were quantified by densitometric analysis of scanned gels using the NIH ImageJ software. For immunoblotting, proteins in isolated mitochondria and/or cytoplasm were separated by SDS-PAGE under reducing conditions, transferred to nitrocellulose membrane, probed with various antibodies raised in rabbits (anti-Aco1, 1:2000; anti-Put2, 1:2000; anti-Nfs1, 1:2000; anti-Dre2, 1:1000), or anti-Pgk1 antibody raised in mouse (1:1000; Molecular Probes), and washed. After incubation with horseradish peroxidase–conjugated secondary antibodies (donkey anti-rabbit IgG or goat anti-mouse IgG; 1:2000), the membrane was washed and subsequently treated with a chemiluminescence reagent, and the signal was developed on X-ray film (44).

## Data availability

All data are included in the article.

## Supporting information

This article contains supporting information (5, 40, 42, 43, 44, 50, 54, 57).

## Conflict of interest

The authors declare that they have no conflicts of interest with the contents of this article.
