# Supplementary material for: Mitochondrial glutaredoxin Grx5 functions as a central hub for cellular iron-sulfur cluster assembly
Source: J Biol Chem. 2025 Mar 10;301(4):108391. doi: 10.1016/j.jbc.2025.108391 (PMC12004709; doi:10.1016/j.jbc.2025.108391)
Supplement: Supporting information [file mmc1.pdf]

Supporting Information

Mitochondrial glutaredoxin Grx5 functions as a central hub for cellular iron-sulfur cluster assembly

Ashutosh K. Pandey, Jayashree Pain, Pratibha Singh, Andrew Dancis, and Debkumar Pain

From the Department of Pharmacology, Physiology and Neuroscience, New Jersey Medical School, Rutgers University, Newark, New Jersey, USA

Table of contents:

1. Figure S1. Characterizing mitochondria and cytoplasm isolated from wild-type cells

2. Figure S2. Activity and Fe-S cluster loading of endogenous aconitase in isolated mitochondria (similar to Fig. 1)

3. Figure S3. Aconitase (Aco1) and cysteine desulfurase (Nfs1) protein levels in various mitochondria

4. Figure S4. Bacterial expression of Grx5 and Isa1 precursor proteins

5. Figure S5. Grx5 precursor protein import into isolated mitochondria

6. Figure S6. Mitochondrial protein import but not Fe-S cluster assembly requires membrane potential

7. Figure S7. Requirement of mitochondria for cytoplasmic Fe-S cluster assembly

8. Table S1. List of yeast strains used in this study

9. References

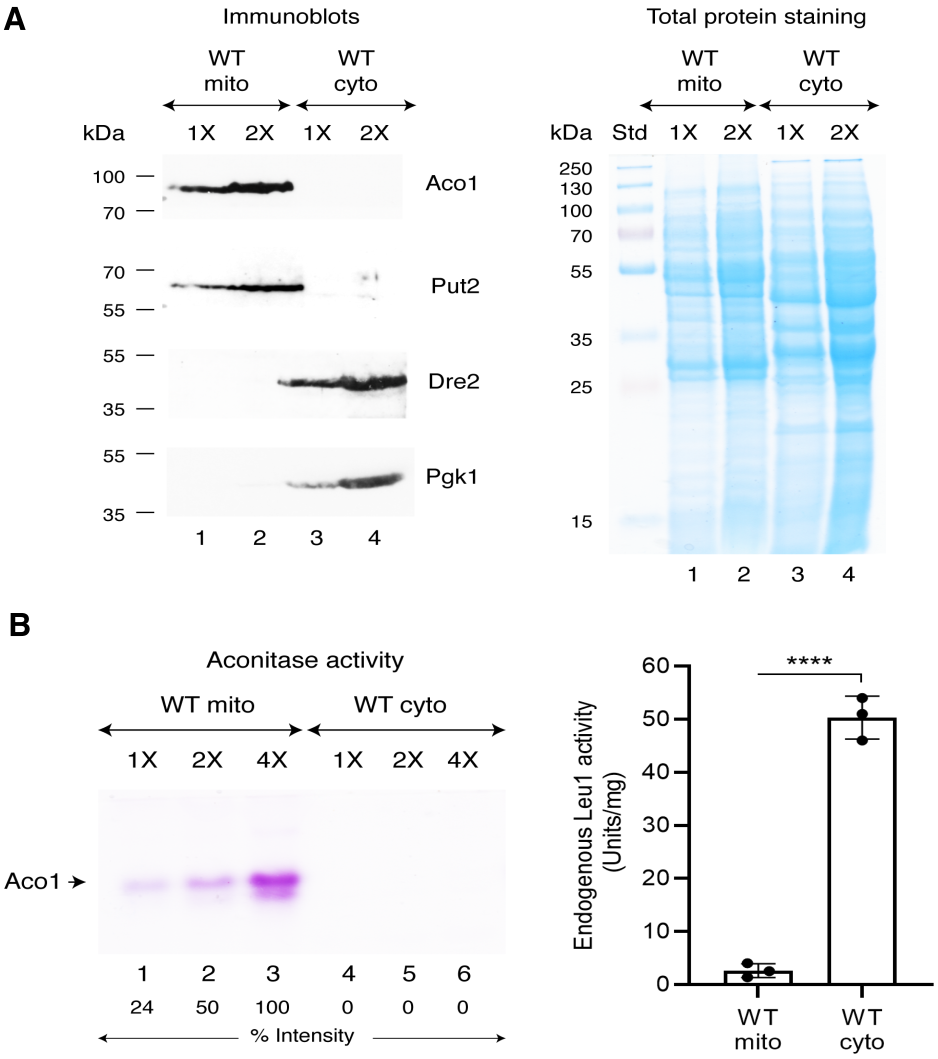

Figure S1. Characterizing mitochondria and cytoplasm isolated from wild-type cells. *A*, immunoblots and total protein staining. Samples containing isolated wild-type mitochondria ("WT mito") or wild-type cytoplasm ("WT cyto") were analyzed by SDS-PAGE, followed by immunoblotting using anti-Aco1, anti-Put2, anti-Dre2, and anti-Pgk1 antibodies as indicated (left panel). In parallel, identical protein samples were run on a separate SDS gel and stained with Coomassie Blue (right panel), serving as loading control for the immunoblots. 1X = 100 µg of proteins. The molecular mass of pre-stained protein standards (Std) is indicated in kDa (left and right panels). *B*, enzyme activity. WT mito and WT cyto samples were subjected to native PAGE and analyzed for aconitase activity by an in-gel assay (1,2). 1X = 25 µg of proteins (left panel). Similarly, a spectrophotometric assay was used to measure endogenous Leu1 isopropylmalate isomerase activity in isolated WT mito and WT cyto samples containing 100 µg of proteins (right panel) (1,3).

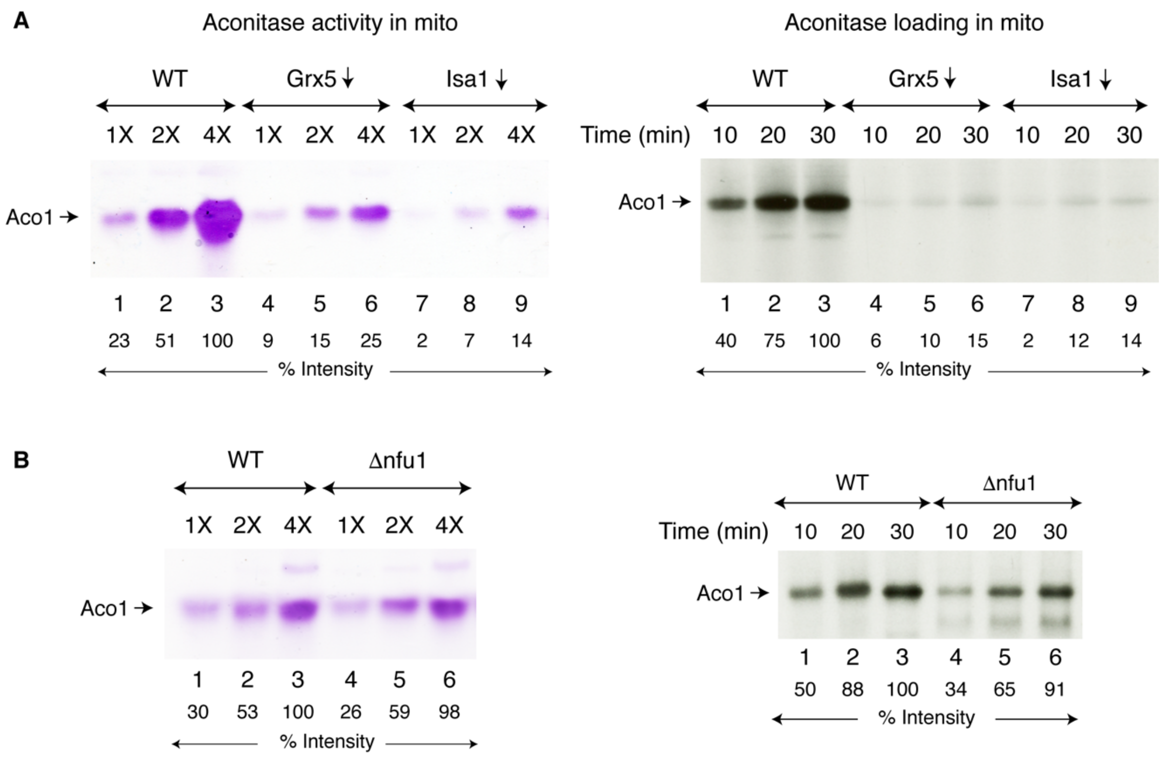

Figure S2. Activity and Fe-S cluster loading of endogenous aconitase in isolated mitochondria (similar to Fig. 1). *A* and *B*, left panels: Aconitase activity. As indicated, mitochondria ("mito") were lysed, subjected to native PAGE, and analyzed for aconitase activity by an in-gel assay (1,2). 1X = 50 µg of proteins. *A* and *B*, right panels: Aconitase loading. WT or mutant mitochondria (200 µg of proteins) were incubated with [^{35}S]cysteine (10 µCi), nucleotides (4 mM ATP, 1 mM GTP, 2 mM NADH), and ferrous ascorbate (10 µM) at 30˚C for 10-30 min. Reaction mixtures were diluted with isotonic buffer and centrifuged. The mitochondrial pellets thus obtained were analyzed by native PAGE, followed by autoradiography (4,5).

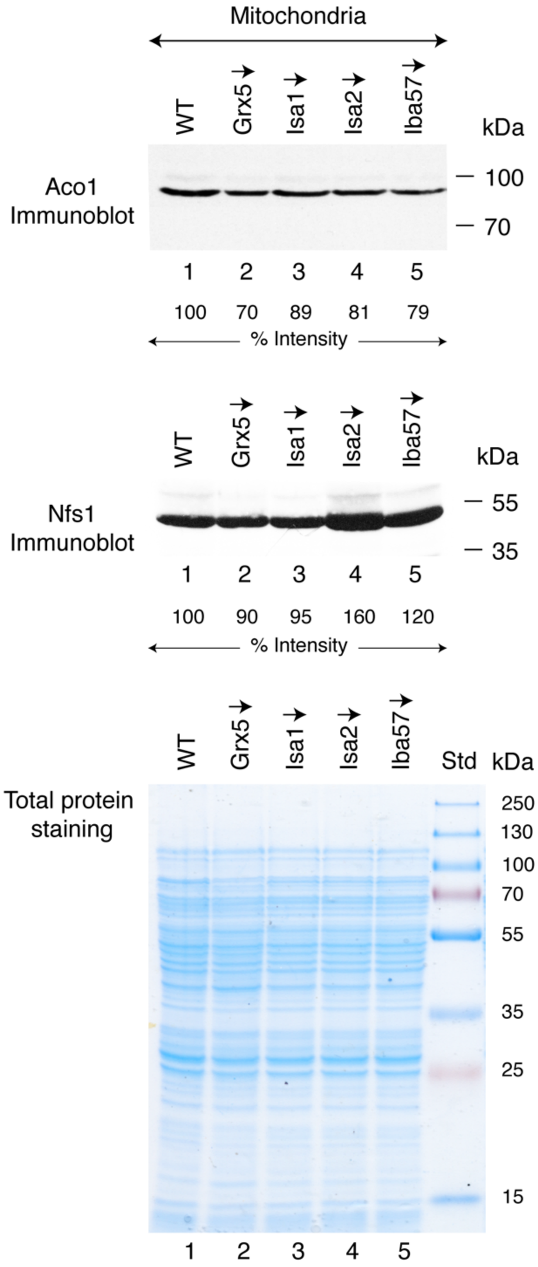

Figure S3. Aconitase (Aco1) and cysteine desulfurase (Nfs1) protein levels in various mitochondria. Mitochondria were isolated from wild-type (WT) and depleted (↓) strains as indicated. Mitochondrial proteins were separated by SDS-PAGE and subsequently analyzed by immunoblotting using anti-Aco1 (top panel) and anti-Nfs1 (middle panel) antibodies. In parallel, identical protein samples were run on a separate SDS gel and stained with Coomassie Blue (bottom panel), serving as loading control for the immunoblots. The molecular mass of pre-stained protein standards (“Std”) is indicated in kDa.

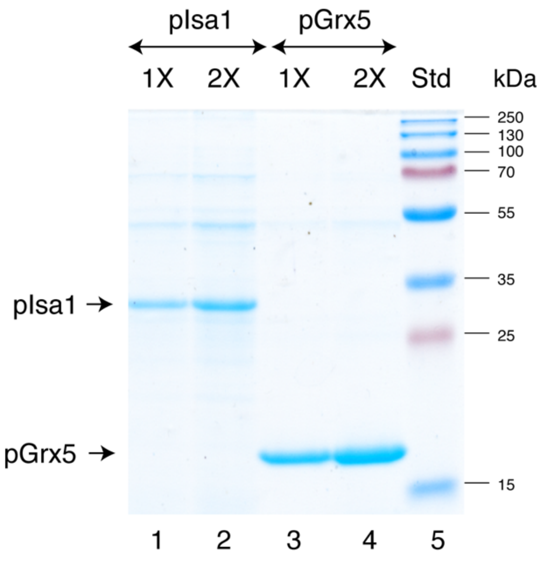

Figure S4. Bacterial expression of Grx5 and Isa1 precursor proteins. The precursor forms of Grx5 (pGrx5) and Isa1 (pIsa1) proteins were individually expressed in BL21 (DE3) cells carrying the plasmid pET21b/pGrx5-His_6 or pET21b/pIsa1-His_6, respectively. The proteins were found to be sequestered in inclusion bodies. The proteins were solubilized with 8 M urea in 50 mM Tris/HCl, pH 8.0, and centrifuged at 250,000 x *g* for 20 min at 20˚C. The supernatant fractions were analyzed by SDS-PAGE, followed by Coomassie Blue staining of the gel. The molecular mass of pre-stained protein standards ("Std") is indicated in kDa. For pIsa1, 1X = 1 µg of protein. For Grx5, 1X = 2 µg of proteins.

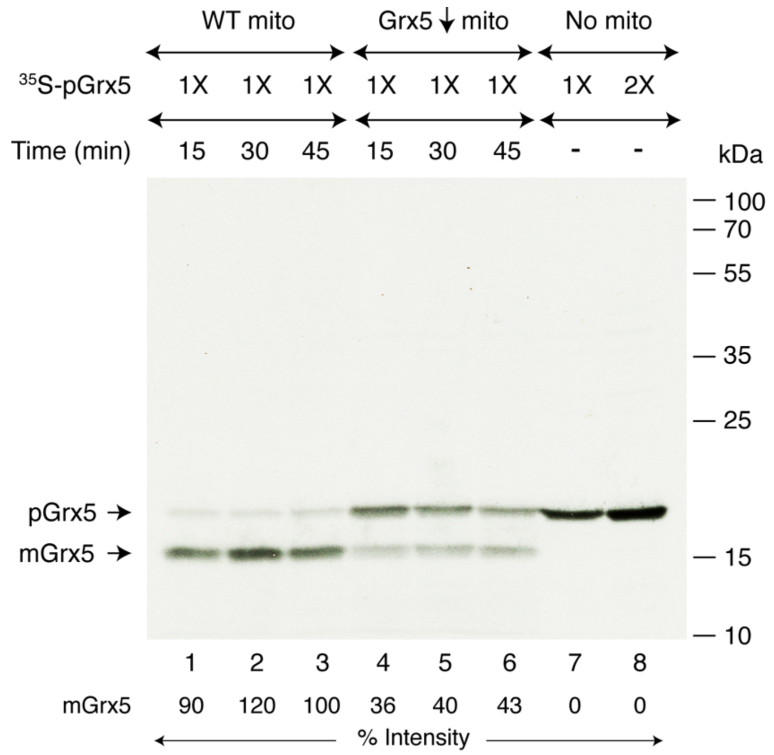

Figure S5. Grx5 precursor protein import into isolated mitochondria. BL21 (DE3) cells carrying the plasmid pET21b/pGrx5-His$_6$ were induced with 1 mM IPTG in M9 media containing 100 μg/ml ampicillin and 10 μCi/ml EXPRESS^{35}S at 37˚C for 3 h. Inclusion body containing ^{35}S-labeled pGrx5 was solubilized with 8 M urea and centrifuged to remove insoluble material, if any. The supernatant containing ^{35}S-pGrx5 was used for the import assay as follows. Isolated mitochondria (WT or Grx5↓; 200 μg of proteins) were supplemented with ATP (4 mM), GTP (1 mM), and NADH (2 mM). Following addition of ^{35}S-pGrx5 (0.5 μg), samples were incubated at 30˚C for 15-45 min. The final urea concentration in the assay mixture was 160 mM. After incubation, samples were diluted with isotonic buffer and centrifuged to reisolate mitochondria. The mitochondrial pellets were analyzed by SDS-PAGE, followed by autoradiography.
1X = 0.25 μg of the ^{35}S-pGrx5 protein used in the assay; mGrx5, mature form of radiolabeled Grx5 generated as a result of ^{35}S-pGrx5 import and removal of the mitochondrial targeting signal (6).

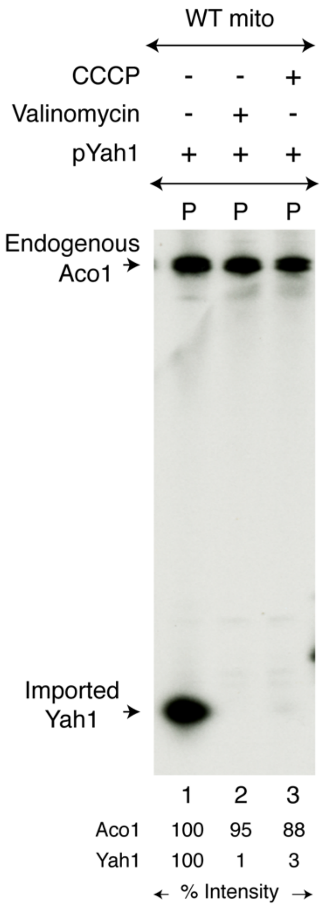

Figure S6. Mitochondrial protein import but not Fe-S cluster assembly requires membrane potential. The precursor form of ferredoxin (pYah1) was expressed in BL21 (DE3) cells carrying the plasmid pET21b/pYah1-His_6. The inclusion body containing the overexpressed pYah1 protein (unlabeled) was solubilized with 8 M urea in 50 mM Tris/HCl, pH 8.0, and centrifuged at 250,000 x *g* for 20 min at 20˚C. The supernatant containing pYah1 was >90% homogenous (4) and was used with no further purification. As indicated, isolated WT mitochondria (200 μg of proteins) were incubated with CCCP (5 μM) or valinomycin (5 μM) for 2 min on ice. Samples were then supplemented with [^{35}S]cysteine (10 μCi), nucleotides (4 mM ATP, 1 mM GTP, 2 mM NADH), ferrous ascorbate (10 μM), and unlabeled pYah1 protein (1 μg). The final urea concentration in the assay mixture was 160 mM. Samples were incubated at 30˚C for 30 min and centrifuged. The mitochondrial pellets were analyzed by native PAGE, followed by autoradiography. In the presence of valinomycin (lane 2) or CCCP (lane 3), mitochondrial membrane potential was dissipated and pYah1 was not imported into mitochondria. Consequently, no imported Yah1 with Fe-^{35}S labeled clusters was detected. Mitochondrial Fe-^{35}S cluster assembly/loading of endogenous aconitase (Aco1) occurred independent of membrane potential (7).

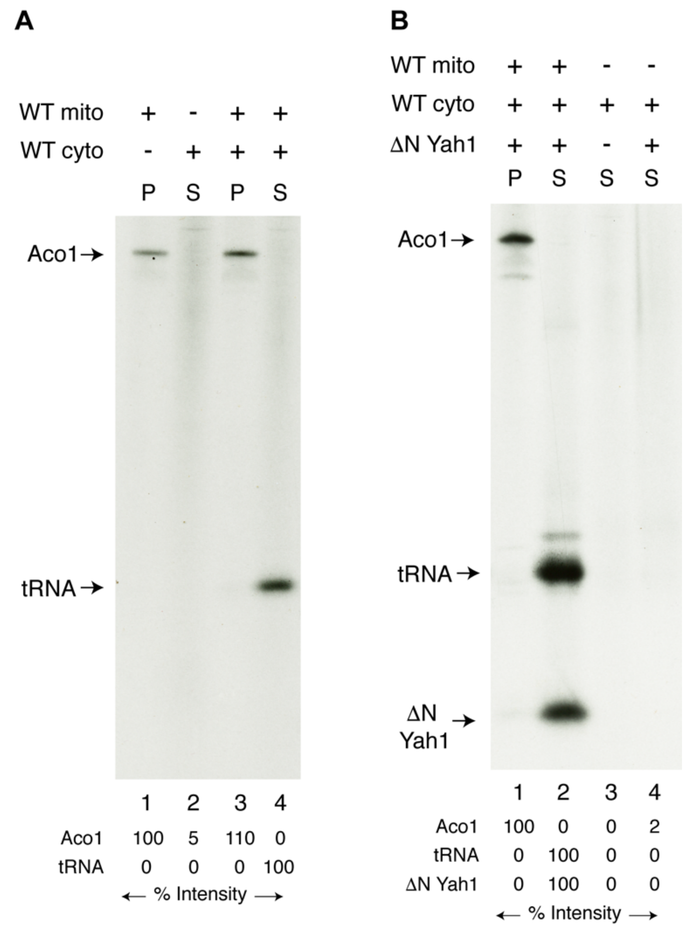

Figure S7. Requirement of mitochondria for cytoplasmic Fe-S cluster assembly. *A*, wild-type mitochondria (WT mito; 200 μg of proteins) alone, wild-type cytoplasm (WT cyto; 200 μg of proteins) alone, or both were mixed with [^{35}S]cysteine (10 μCi), nucleotides (4 mM ATP, 1 mM GTP, 2 mM NADH), and ferrous ascorbate (10 μM). Samples were incubated at 30˚C for 30 min and then centrifuged. The resulting mitochondrial pellet ("P") and cytoplasm/supernatant ("S") fractions were analyzed by native PAGE, followed by autoradiography (5). *B*, as indicated, reaction mixtures were assembled with WT mitochondria (200 μg of proteins), WT cytoplasm (200 μg of proteins), and apo-ΔNYah1 protein (1 μg). All samples were supplemented with [^{35}S]cysteine, nucleotides, and ferrous ascorbate. After incubation at 30˚C for 30 min, samples were centrifuged and as indicated, mitochondrial pellet ("P") and/or cytoplasm/supernatant ("S") fractions were analyzed as in (*A*) above (5).

Table S1. List of yeast strains used in this study.

Number	Strain name	Genotype	Source
DP 547	BY4741 (WT)	MATa his3Δ1 leu2Δ0 met15Δ0 ura3Δ0	(4,5)
DP 3538	Δleu1	MATa his3Δ1 leu2Δ0 met15Δ0 ura3Δ0 Δleu1::KanMX6	Open Biosystems
DP 3239	nfs1-14 (nfs1)	MATa ura3-52 lys2-801 (amber) ade2-101 (ochre) trp1-Δ63 his3-Δ200 nfs1-LEU2::LEU2 cyh2	(5,8)
DP 3457	Gal-Ssq1	MATa his3Δ1 leu2Δ0 met15Δ0 ura3Δ0 His3MX6-PGAL1-SSQ1::SSQ1	(5)
DP 3505	Gal-Grx5	MATa his3Δ1 leu2Δ0 met15Δ0 ura3Δ0 His3MX6-PGAL1-GRX5::GRX5	This work
DP 3504	Gal-Isa1	MATa his3Δ1 leu2Δ0 met15Δ0 ura3Δ0 His3MX6-PGAL1-ISA1::ISA1	This work
DP 3508	Gal-Isa2	MATa his3Δ1 leu2Δ0 met15Δ0 ura3Δ0 His3MX6-PGAL1-ISA2::ISA2	This work
DP 3509	Gal-Iba57	MATa his3Δ1 leu2Δ0 met15Δ0 ura3Δ0 His3MX6-PGAL1-IBA57::IBA57	This work
DP 3472	Δnfu1	MATa his3Δ1 leu2Δ0 met15Δ0 ura3Δ0 Δnfu1::KanMX6	Open Biosystems
DP 3473	Δbol3	MATa his3Δ1 leu2Δ0 met15Δ0 ura3Δ0 Δbol3::KanMX6	Open Biosystems

References

1. Pandey, A. K., Yoon, H., Pain, J., Dancis, A., and Pain, D. (2024) Mitochondrial acyl carrier protein, Acp1, required for iron-sulfur cluster assembly in mitochondria and cytoplasm in Saccharomyces cerevisiae. *Mitochondrion* **79**, 101955
2. Pandey, A., Pain, J., Dziuba, N., Pandey, A. K., Dancis, A., Lindahl, P. A., and Pain, D. (2018) Mitochondria Export Sulfur Species Required for Cytosolic tRNA Thiolation. *Cell Chem. Biol.* **25**, 738-748
3. Pandey, A. K., Pain, J., Brindha, J., Dancis, A., and Pain, D. (2023) Essential mitochondrial role in iron-sulfur cluster assembly of the cytoplasmic isopropylmalate isomerase Leu1 in *Saccharomyces cerevisiae*. *Mitochondrion* **69**, 104-115
4. Amutha, B., Gordon, D. M., Gu, Y., Lyver, E. R., Dancis, A., and Pain, D. (2008) GTP is required for iron-sulfur cluster biogenesis in mitochondria. *J. Biol. Chem.* **283**, 1362-1371
5. Pandey, A. K., Pain, J., Dancis, A., and Pain, D. (2019) Mitochondria export iron-sulfur and sulfur intermediates to the cytoplasm for iron-sulfur cluster assembly and tRNA thiolation in yeast. *J. Biol. Chem.* **294**, 9489-9502
6. Rodríguez-Manzaneque, M. T., Tamarit, J., Bellí, G., Ros, J., and Herrero, E. (2002) Grx5 is a mitochondrial glutaredoxin required for the activity of iron/sulfur enzymes. *Mol. Biol. Cell* **13**, 1109-1121
7. Pain, J., Balamurali, M. M., Dancis, A., and Pain, D. (2010) Mitochondrial NADH kinase, Pos5p, is required for efficient iron-sulfur cluster biogenesis in *Saccharomyces cerevisiae*. *J. Biol. Chem.* **285**, 39409-39424
8. Li, J., Kogan, M., Knight, S. A., Pain, D., and Dancis, A. (1999) Yeast mitochondrial protein, Nfs1p, coordinately regulates iron-sulfur cluster proteins, cellular iron uptake, and iron distribution. *J. Biol. Chem.* **274**, 33025-33034
